# Pharmaco-toxicological aspects of thymol in veterinary medicine. A systematic review

**DOI:** 10.3389/fvets.2025.1562641

**Published:** 2025-08-12

**Authors:** Denisa Pérez Gaudio, Mery Giantin, Marianna Pauletto, Mauro Dacasto

**Affiliations:** ^1^Laboratorio de Toxicología, Depto. de Fisiopatología, Facultad de Ciencias Veterinarias, Centro de Investigación Veterinaria de Tandil (CIVETAN), Universidad Nacional del Centro de la Provincia de Buenos Aires, Tandil, Buenos Aires, Argentina; ^2^Consejo Nacional de Investigaciones Científicas y Técnicas (CONICET), Buenos Aires, Argentina; ^3^Department of Comparative Biomedicine and Food Science, Division of Veterinary Pharmacology and Toxicology, University of Padua, Padua, Italy

**Keywords:** thymol, pharmacological effects, toxicological effects, veterinary applications, *in vivo*, *in vitro* and *in silico* studies

## Abstract

**Introduction:**

Thymol, a phenolic compound present in essential oils, has shown promising pharmacological properties and potential veterinary applications. This systematic review aims to evaluate the pharmacological and toxicological effects of thymol on animals.

**Materials and methods:**

A comprehensive search following PRISMA 2020 guidelines was conducted across databases including PubMed, Scopus, Google Scholar, Web of Science, and LILACS. Various health descriptors, medical subject headings terms, and their synonyms were used to identify studies examining thymol's effects in animals, including its *in vitro, in vivo*, and *in silico* toxicity, as well as its possible environmental impact. Only relevant experimental studies from the last 12 years were included, provided they assessed thymol itself, one of its primary phytoadditive sources, or a blend where thymol was at least as concentrated as other components. The protocol was registered in Open Science Framework (DOI: https://doi.org/10.17605/OSF.IO/B6SF9).

**Results:**

A total of 1.472 records were identified, with 176 meeting inclusion criteria. Studies spanned from 2012 to 2024, indicating that thymol exhibits antimicrobial, antiviral, antifungal, antiparasitic, anti-inflammatory, and antioxidant properties, among others. However, thymol displays dose-dependent toxicity, especially at high levels, affecting mainly the liver and kidneys.

**Discussion:**

Thymol holds substantial potential as a therapeutic agent in veterinary medicine due to its beneficial effects and relatively low toxicity. Nonetheless, further research is needed to establish safe and effective dosages across different animal species.

**Systematic review registration:**

https://doi.org/10.17605/OSF.IO/B6SF9.

## 1 Introduction

**Figure 1:**
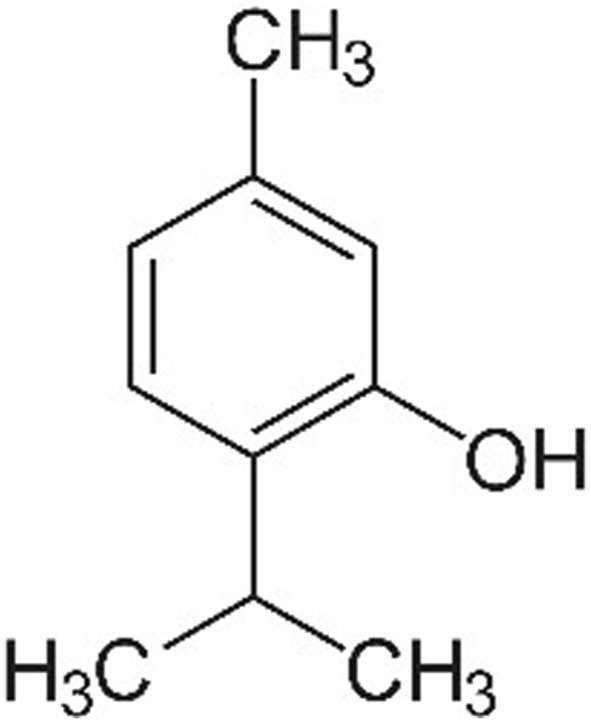
Thymol's chemical structure.

The use of natural compounds in veterinary medicine has garnered increasing interest as alternatives to conventional pharmaceuticals, driven by concerns over antimicrobial resistance and the potential side effects of synthetic drugs. Among these natural compounds, thymol (THY; 2-isopropyl-5-methylphenol), a monoterpenoid phenol (Figure 1), has been widely studied for its pharmacological properties across various animal species, including both production and companion animals. THY is primarily found in plants of the *Thymus* genus, especially *Thymus vulgaris* (common thyme), but it is also present in *Origanum* (oregano), *Ocimum* (basil), *Monarda* species (bergamot, bee balm, horsemint, and Oswego tea), *Lippia origanoides* (Mexican oregano), and *Nigella* species (e.g., *Nigella sativa*, black cumin), among others (1). Given its presence in several essential oils (EO), THY is often incorporated into phytotherapeutic blends, where it exhibits synergistic or additive effects when combined with other active constituents, such as carvacrol (CAR), eugenol (EUG), and cinnamaldeyde (CIN), to enhance its efficacy and expand its range of biological activities (2–6).

THY has demonstrated a range of pharmacologically beneficial activities that are highly relevant in veterinary contexts. Studies have consistently reported its antimicrobial, antifungal, anti-inflammatory, antioxidant, and analgesic properties, making it a promising candidate for managing various health conditions in animals (1). THY has also demonstrated anticancer effects in human cell lines; however, studies in animal models are scarce, and further research in broader and more specific animal systems is required to establish its potential utility in veterinary oncology (7).

Notably, THY has been shown to exert broad-spectrum antimicrobial activity against both Gram-positive and Gram-negative bacteria of production and companion animals, which is of particular importance in veterinary applications where zoonotic pathogens are common. In small animals, THY's antimicrobial effect has potential applications, particularly for oral and dermatological care. THY-containing products are widely used in pet oral care formulations, which help reduce dental plaque and prevent periodontal diseases, conditions prevalent among pets (8). In these species, THY and *Thymus vulgaris* essential oils (THEO) have also exhibited notable antiviral activity against a variety of viruses, including feline calicivirus (FCV), feline coronavirus (FCoV), and norovirus surrogates. Moreover, its antifungal activity offers potential in treating or preventing infections caused by pathogens such as *Pythium* (9) and *Aspergillus* species (10). In large animals, THY has also been shown to be promising as both an internal and external antiparasitic agent, effectively targeting gastrointestinal parasites as well as ectoparasites like ticks and mites, which contributes to improved animal health and productivity without relying on conventional synthetic antiparasitics (11). On the other hand, in companion animals, THY has also been investigated as an ectoparasiticide, showing potential in controlling flea and tick infestations without resorting to synthetic insecticides that may have adverse effects on pets and the environment (12). Such applications underscore the versatility of THY in veterinary care, making it an attractive option for a wide range of therapeutic interventions across multiple species.

The anti-inflammatory and antioxidant properties of THY have also been explored, particularly concerning its role in modulating immune responses and reducing oxidative stress, which can significantly impact animal health, especially in intensive production systems (13, 14). These properties suggest that THY could serve as a supportive therapy in managing chronic inflammatory conditions or as a preventive measure against stress-induced pathologies (15), common in both livestock and companion animals.

In production animals, THY has been extensively studied as a feed additive aimed at promoting growth and improving gut health. THY is known to influence gut microbiota composition positively (16, 17), enhancing the populations of beneficial microbes while inhibiting pathogenic bacteria, which results in better nutrient absorption and overall health (18). The inclusion of THY in livestock diets has been shown to improve weight gain, feed efficiency, and immune function, particularly in poultry and swine production (19, 20). These benefits have driven interest in THY as a natural growth promoter, especially as the use of antibiotic growth promoters has been increasingly restricted in various regions due to concerns over antibiotic resistance (21).

Furthermore, THY-containing blends that combine other EO demonstrated enhanced efficacy in promoting health and productivity in ruminants, such as cattle and sheep, by stimulating ruminal fermentation and enhancing fiber digestion (22). Such applications have shown promise in reducing methane emissions, a relevant environmental consideration, thereby contributing to more sustainable livestock production.

Research in laboratory animals, particularly rodents, is essential in veterinary pharmaco-toxicology for understanding compounds like THY. Rodent studies provide key data on THY's pharmacokinetics [absorption, distribution, metabolism, and excretion (ADME)] and help define dosing protocols for larger animals. Toxicological assessments in rats also clarify safety thresholds, reduce risks in veterinary applications, and reveal mechanisms of action that often translate to other species. Furthermore, these models highlight potential metabolic interactions relevant to long-term use or concurrent administration with other agents (23).

THY has also been extensively studied in cell cultures of animal origin to elucidate its pharmacological effects at the cellular level, especially regarding its antimicrobial, anti-inflammatory, and antioxidant properties. *In vitro* studies using animal-derived cells, such as intestinal and hepatic cell lines, demonstrated THY's ability to modulate cellular responses to oxidative stress and inflammation, mechanisms that are central to maintaining tissue integrity in both production and companion animals (24–26). Additionally, THY has shown cytotoxic effects on specific pathogenic organisms when applied to cell cultures, highlighting its potential as a natural antimicrobial agent in veterinary medicine (27). These cellular models revealed THY's dose-dependent effects, providing critical insights into its therapeutic thresholds and cytotoxic potential, which are essential for safe and effective application in animal health.

This systematic review aims to provide a comprehensive analysis of the pharmaco-toxicological properties of THY in veterinary medicine by synthesizing evidence from studies conducted on production animals, companion animals, and, where relevant, laboratory rats and mice. By evaluating the efficacy, safety, and potential limitations of THY, both as a standalone compound and in phytotherapeutic blends, this review seeks to inform veterinary practitioners about THY's applicability and contribute to evidence-based guidelines for its use. Given the increasing restrictions on synthetic antimicrobials and growth promoters, understanding the potential of THY and similar compounds is crucial for developing sustainable, effective, and safe alternatives in veterinary care.

## 2 Materials and methods

### 2.1 Question and PICOS strategy

This systematic review focuses on the toxicological and pharmacological aspects of THY and its main phytochemical sources in production and companion animals, and its purpose is to answer the following questions: Is THY toxic for animal cells and tissues? Which beneficial effect does THY exhibit on animal cells (i*n vitro*), animal models of diseases, animal physiology or different pathologies (*in vivo*)? The review followed the guidelines of the Preferred Reporting Items for Systematic Reviews and Meta-Analyses (PRISMA) (28). Moreover, a PICOS strategy (patient or pathology, intervention, control, and other outcomes and type of study) was used based on: P: healthy or microorganism challenged animals; I: treatment with THY; C: no treatment, healthy cells or animals receiving placebo (vehicle) or absence of THY source; O: cytotoxic and beneficial effects; S: *in vitro* and *in vivo* studies.

### 2.2 Data sources and literature search

The literature search was conducted on the Web of Science, PubMed, Scopus, LILACS, and Google Scholar until December 2024. A combination of search terms was used, structured with Boolean operators to ensure comprehensive coverage. Keywords included thymol-related terms (“thymol” OR “essential oils” OR “thyme extract”), pharmacological descriptors (“pharmacological” OR “antioxidant” OR “antimicrobial” OR “bioactivity”), toxicological descriptors (“toxicity” OR “cytotoxicity” OR “safety”), pharmacokinetic-related terms (“pharmacokinetics” OR “pharmacodynamics” OR “absorption” OR “distribution” OR “metabolism” OR “excretion” OR “ADME”), and species-specific terms (“rats” OR “cattle” OR “pigs” OR “chickens” OR “rabbits”), among others. These groups of terms were combined using the AND operator to retrieve studies addressing thymol or its sources, their biological effects, and relevance to veterinary species.

Given the broad range of databases, studies, and species, the search strategy was designed to maximize coverage while maintaining relevance to the research questions. This ensured that key studies across diverse models and different animal species were included. Articles published between 2012 and 2024 were considered to ensure the inclusion of the most current and relevant research on THY's toxicological and pharmacological properties. The initial search was then followed by manual screening of reference lists from selected studies to capture any additional articles not indexed in the databases.

### 2.3 Study selection

**Figure 2:**
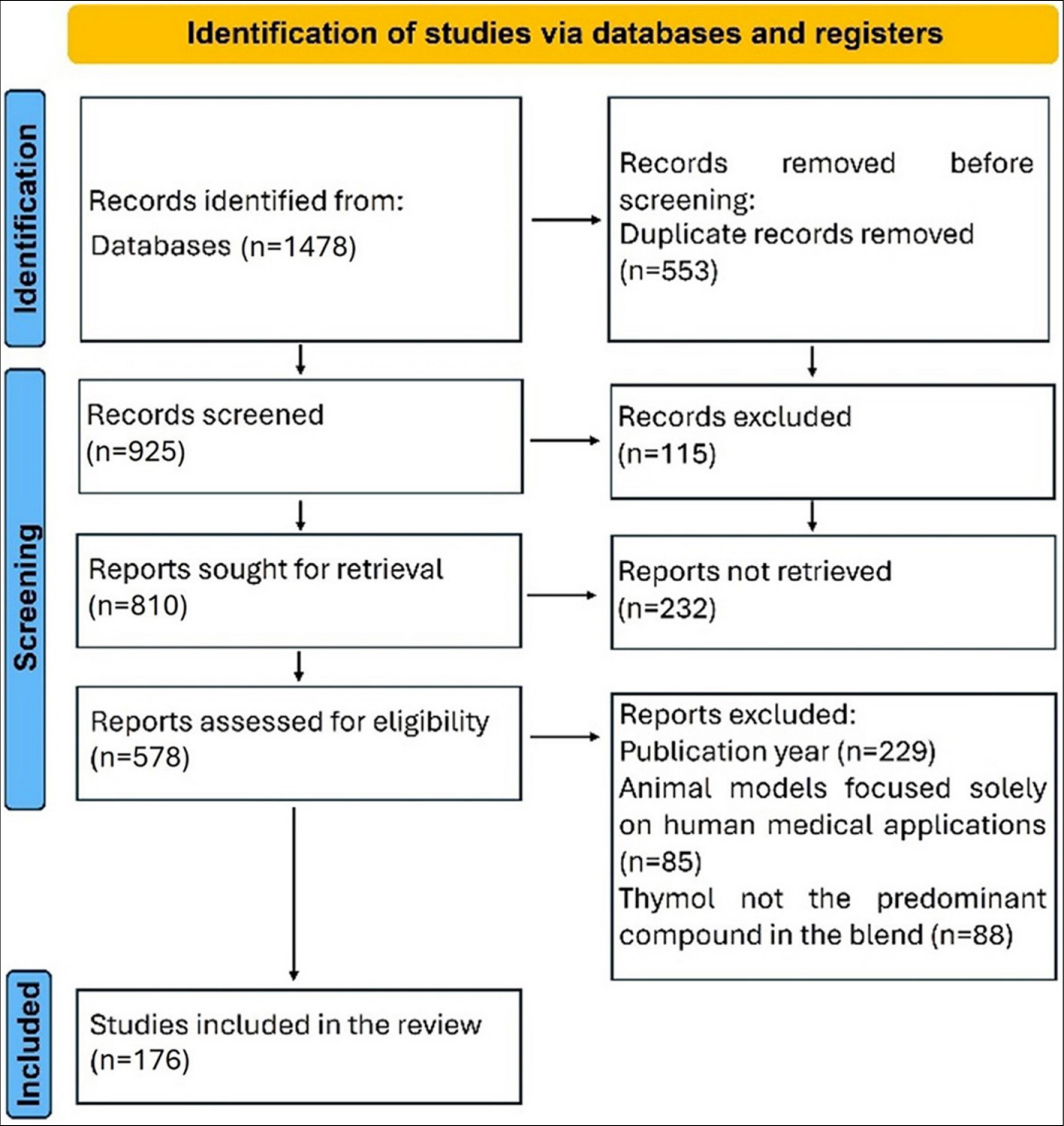
A flow diagram is used for the selection of the studies included in the systematic review.

The inclusion criteria for the systematic review were as follows: (i) all types of investigation design (*in vitro* and *in vivo* studies, including murine models of pathologies relevant to veterinary medicine); (ii) studies related specifically to the application of THY, or a phytoadditive THY source where THY was the main component or when the exact composition is not reported by authors but it is also known that THY is the main component of the plant, or the use of a blend where THY concentration was superior or equal to the other components; (iii) manuscripts that assessed directly or indirectly the toxicological or beneficial effects of THY in companion animals, production animals (conventional and non-conventional), and other species of veterinary interest. For article exclusion, the following specific criteria were defined: (i) restrictions on year of publication, selecting only articles published in the period 2012–2024; (ii) articles using mice, rats, or cells from murine origin were excluded if they were used as a model for exclusive human diseases and their results could not be of importance for animal treatments; (iii) studies involving THEO or blends in which THY was included but not as the primary compound. Figure 2 shows the flow diagram used for the selection of the studies included in the systematic review.

### 2.4 Data extraction

Data extraction was carried out by two independent reviewers using a standardized form. The form collected key information, including study design, animal species, doses of THY or THY-based additives, assessment of toxicity (cytotoxicity, organ toxicity, or adverse effects), and beneficial effects (e.g., antioxidant, antimicrobial, and immunomodulatory). Discrepancies between reviewers were resolved through discussion. The data extracted from each study were then organized and transformed when necessary to ensure consistency across studies, particularly regarding units of measurement.

### 2.5 Quality assessment and risk of bias

The quality of the studies included in the systematic review was assessed using predefined criteria, which evaluated the methodological rigor of each study. Key factors, such as randomization, blinding, control groups, and potential sources of bias, were considered. Studies that were judged to have a high risk of bias or those that lacked sufficient methodological transparency were excluded or treated with caution in the analysis. This process ensures that the final synthesis is based on studies with reliable and robust evidence. The risk of bias and quality assessment was performed independently by two reviewers, with any discrepancies resolved by discussion.

### 2.6 Synthesis and presentation of results

The results of the included studies were synthesized using both tabular and narrative approaches. Key information, including study characteristics, types of interventions, outcomes measured, and major findings, was systematically organized and presented in summary tables to facilitate comparison across studies.

The consistency of the results across studies was assessed qualitatively, considering the strength, direction, and relevance of the observed effects.

Subsequently, the discussion section was developed through a comprehensive narrative integration of the findings, highlighting patterns, discrepancies, and the overall interpretation of the evidence within the context of veterinary medicine.

Due to the high degree of methodological and outcome variability among studies, no meta-analysis was performed.

### 2.7 Protocol registration

This systematic review was prospectively registered in the Open Science Framework (OSF) under the title *Pharmaco-toxicological aspects of thymol in veterinary medicine. A systematic review*, with the following DOI: https://doi.org/10.17605/OSF.IO/B6SF9. The registration includes the review protocol, eligibility criteria, and synthesis strategy to ensure transparency and reproducibility of the research process.

## 3 Results

**Figure 3:**
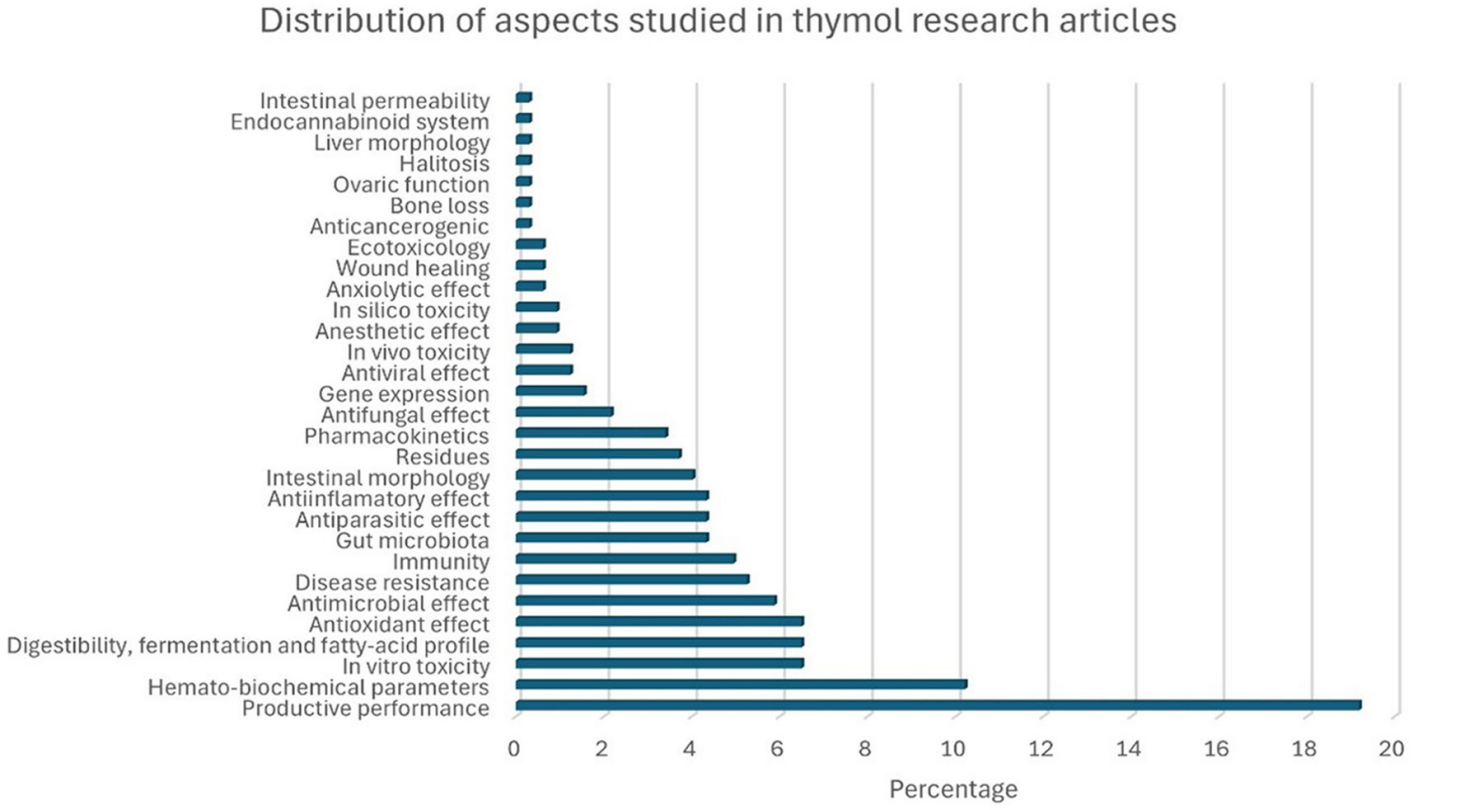
Percentage distribution of research articles by aspect of THY studied.

**Figure 4:**
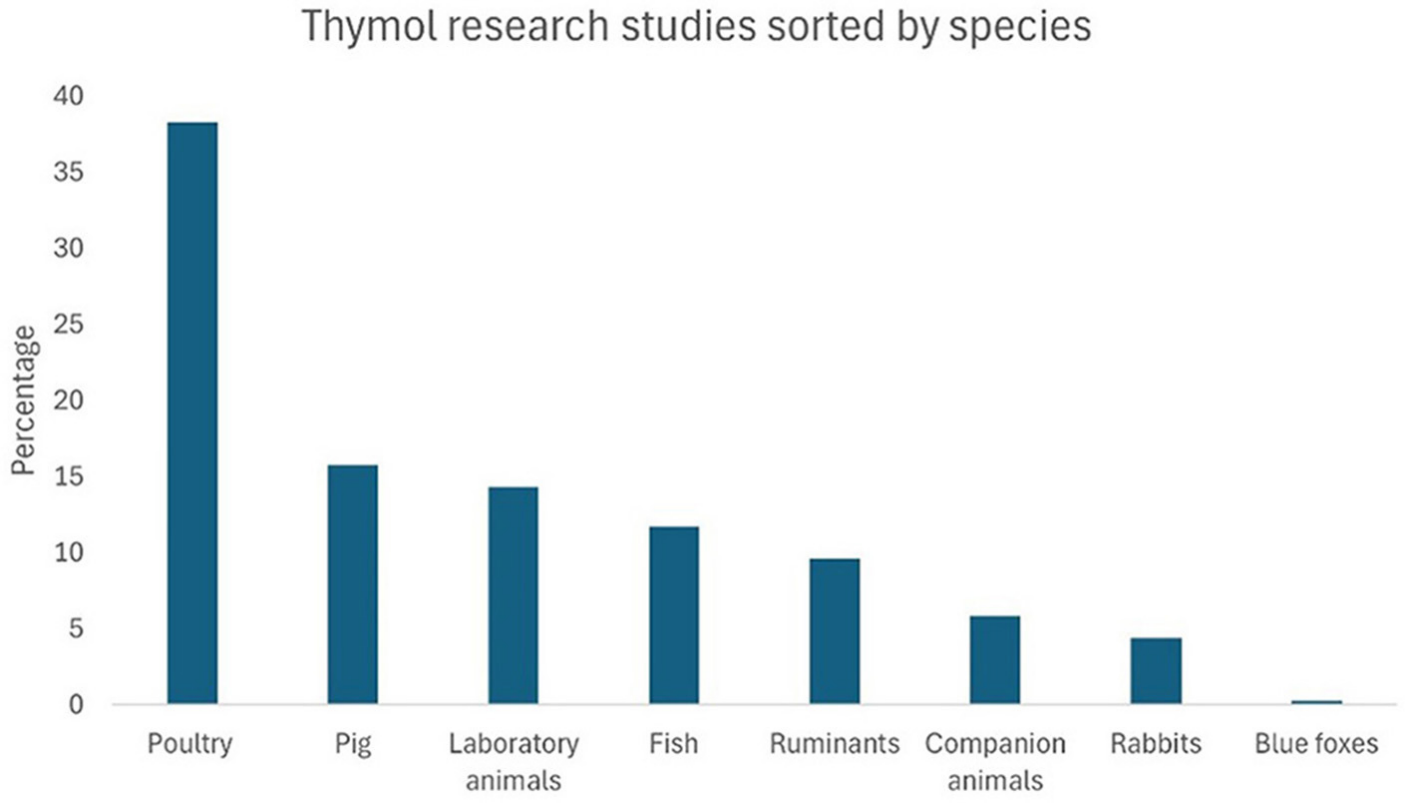
Percentage distribution of THY research articles by species.

A total of 1,472 records were obtained, of which 176 met the inclusion criteria for this systematic review. Figure 3 shows a bar chart illustrating the distribution of the research articles included, based on the aspects of THY that were studied. “Productive performance” was the most frequently examined aspect, accounting for 19.2% of the articles, followed by “hemato-biochemical” effects (10.2%). Other commonly researched areas included “antioxidant” properties, “digestibility, fermentation, fatty-acid profile,” and “*in vitro* toxicity,” each representing ~6.5% of the studies. Less studied aspects, such as “intestinal permeability,” “liver morphology,” and “halitosis” accounted for <1% of the total. This distribution highlights a primary focus on productivity and biochemical impacts of THY in animal studies, with fewer studies addressing niche areas. Figure 4 presents THY research studies grouped by species: poultry (broiler chickens, quails, laying hens, ducks), pigs, laboratory animals (rats and mice), fish (tilapia, rainbow trout, and common carp), ruminants (cattle, sheep, and goats), companion animals (dogs and cats), rabbits, and blue foxes. Studies performed with cell lines, primary cell cultures, and pathogens from a particular species were also included.

### 3.1 THY toxicity

#### 3.1.1 Cytotoxicity

Table 1 summarizes studies on the cytotoxicity of THY and its derivatives in different cell lines. This table presents the cell types used, the concentrations of THY or THEO to which they were exposed, the methods applied to evaluate cell viability, as well as the specific values of cytotoxicity or effects observed (e.g., LC_50_, IC_50_, CC_20_). The results revealed variability in the cytotoxic response, depending on the cell type and concentrations used.

**Table 1:** THY toxicity in different animal cell lines.

**Cell type**	**Cell culture/cell line and species of origin**	**Assay**	**Exposure**	**Findings**	**References**	**Year**
Enterocytes	Canine BMSC (primary cell culture) and canine BMSC differentiated into ELC cells (primary cell culture)	Alamar blue	THY: 4.66–2,529.67 μM THEO (THY: 40%): 11.32–6,113.89 μM	LC_50_ for THY is 13.32 ± 0.67 μM (BMSC) and 13.32 ± 5.33 μM (ELC) LC_50_ for THEO is 533.06 ± 19.99 μM (BMSC), 393.04 ± 6.66 μM (ELC)	(30)	2015
	IPEC-J2 cells (cell line; pig)	WST-1	THY: 10–200 μM	THY at <100 μM shows no significant effect on viability; at >100 μM, it significantly reduces viability	(25)	2019
		Trypan blue staining	THY-CIN blend: 0.0067–6656.37 μM; 48 h	High viability (>90%) is observed for concentrations up to 6.66 μM	(31)	2020
Hepatocytes	Porcine hepatocytes (primary cell culture)	Not reported	THEO from three different *Thymus* species (THY: 38.5%, 49.10% and 56.02%): >2,663.44 μM	No toxicity is detected for any of the EO tested	(32)	2013
	Canine hepatocytes (primary cell culture)	Alamar blue	THY and THEO (THY: 40%): 0.0665–6.665 μM	LC_50_ for THY is 0.33 ± 0.07 μM LC_50_ for THEO is 1.20 ± 0.16 μM	(33)	2015
	LMH cells (cell line; chicken)	Trypan blue staining	THY-CIN blend (75% 25%); 0.0067 μM a 667 μM; 48 h	High viability (>90%) is observed up to a maximum of 0.67 μM	(34)	2020
Renal Cells	Vero (cell line; African green monkey)	XTT	THY: 25–400 μM; 4 days	CC_50_: 300 μM	(35)	2012
	MDCK (cell line; dog)	BAPTA/AM chelation)	THY: 300–500 μM	Cytotoxicity not prevented by Ca^2+^ chelation with BAPTA/AM	(36)	2014
	MDCK (cell line; dog)	Alamar blue	THY: 0.3–0.06 mg/ml and THEO (THY: 40%): 0.18–0.03 mg/ml	THY is more toxic (LC_50_ = 0.13 ± 0.002 mg/ml) than THEO (LC_50_ = 0.16 ± 0.01 mg/ml)	(37)	2015
	FRhK-4 (cell line; monkey)	Visual inspection under optical microscope	THY: 0.1–2%.	Cytotoxicity is observed at THY 2%.	(38)	2015
	CRFK (cell line; cat)	XTT and visual inspection under optical microscope	THEO (THY: 47%): 8,900, 4,450, 2,225, 1,112.5, 556.25, 278.12, 139.06, 69.53 μg/mL	CC_20_ for THEO: 194.98 μg/mL	(39)	2024
Splenocytes	Rat splenocytes (primary cell culture)	MTT	THY: 0.5–500 μM	IC_50_: 362 μM	(40)	2013
Macrophages	RAW 264.7 cells (cell line; mice)	MTT	THY and derivatives (acetyl-THY and benzoyl-THY): 0–100 μg/mL; 48 h	THY at 100 μg/mL reduces survival to 36.5%; benzoyl-THY at 100 μg/mL decreases macrophage survival to 63.6%, and acetyl-THY at 100 μg/mL shows >100% survival	(3)	2014
		Visual inspection under optical microscope	THY: 0.1–2%.	Cytotoxicity is observed at THY 2%.	(38)	2015
		MTT	THY: 10–40 μg/mL; 24 h	No cytotoxicity is observed at tested doses	(41)	2017
		MTT	THY: 25–400 μM	No cytotoxicity is observed at tested doses	(42)	2018
	RAW 264.7 cells (cell line; mice) and bone marrow macrophages (primary cell culture)	MTT	THY: 10–40 μM; 24 h	No cytotoxicity is observed at tested doses	(26)
Leukocytes	Mice peritoneal leukocytes (primary cell culture)	MTT	THY (1.5–150 μg/mL)	Viability remains >83% at all tested concentrations	(43)	2012
Monocytes	HD11 monocytes (cell line; chicken)	Trypan blue staining	THY-CIN blend (75% 25%; 1–100,000 ng/ml; 48 h	High viability (>90%) is observed	(34)	2020
Peripheral blood mononuclear cells	Cattle PBMC (primary cell culture)	MTT	THY:1 and 10 Mm	No cytotoxicity is observed at tested doses	(44)	2023
Mammary epithelial cells	Mouse mammary epithelial cells (primary cell culture)	MTT	THY: 10–40 μg/mL; 24 h	No cytotoxicity is observed at tested doses	(24)	2014
	Bovine mammary epithelial cells (primary cell culture)	CCK-8	THY: 16–64 mg/mL; 48 h	No cytotoxicity is observed at tested doses	(45)	
Fibroblasts	NIH-3T3 fibroblasts (cell line; mice)	MTT	Enriched cellulose hydrogel with THY: 0.25–1%	Viability decreases slightly with increasing concentration (98.84 to 88.81%); overall, low toxicity is observed	(46)	2018

#### 3.1.2 *In vivo* toxicity

Table 2 presents findings from *in vivo* studies assessing the toxicity of THY and its derivatives in different animal models. The studies highlighted both acute and sub-chronic toxicity data across various dosages, providing insight into lethal dose thresholds, tissue-specific toxic effects, and potential developmental and estrogenic impacts. In murine models, THY exhibited dose-dependent lethality, with THY acetate showing reduced toxicity, suggesting a higher safety margin. Other studies reported THY's influence on immune cells and inflammatory responses, pointing to immunotoxic effects. In chicken embryos, THY exposure at higher concentrations caused specific developmental abnormalities, whereas *in vitro* assays revealed weak estrogenic and mutagenic activities below the established thresholds for genotoxicity.

**Table 2:** *In vivo* toxicity of THY and THY derivatives in different animal models.

**Species**	**Type of study**	**Compound**	**Dosage and administration**	**Observations and results**	**Conclusion**	**References**	**Year**
Mice	Acute toxicity	THY and THY acetate	THY: 0, 250, 500, 1,000, and 2,000 mg/kg THY acetate: 0, 2,000, 2,500, 3,000, and 3,500 mg/kg Single oral dose	THY LD_10_ is 772.1 mg/kg, LD_50_ is 1,350.9 mg/kg THY acetate LD_10_ is 2,522.8 mg/kg, LD_50_ is 4,144.4 mg/kg	THY acetate displays reduced toxicity compared to THY, indicating a higher safety margin	(47)	2017
	Immunotoxicity	THY	0, 10, 20, and 40 mg/kg. 30-day oral dose	THY alters hydrolysis of adenine nucleotides in serum, spleen, and splenic lymphocytes. It downregulates NTPDase (ATP substrate) and upregulates ADA activity, indicating inflammation and immune toxicity	THY's effect on immune response is linked to its toxicological impact on inflammation	(48)	2018
Chronic toxicity	THY	0, 0.1, and 0.5% Spray into a breathing tank (three treatment sessions of 10 min each, per week for 6 months (26 weeks)	No statistically significant differences are found in the chronic toxicity index for the mice that inhaled high doses of THY and those with no THY inhaled	No significant chronic toxicity differences are observed under long-term repeated exposure to high doses of inhaled THY	(49)	2019
Chicken	Embryotoxicity, estrogenic effects and mutagenicity	THY	Embryonic assay: 50 μg/kg and 500 μg/kg; estrogenic and mutagenicity assays: 1.5 μg/L to 150 μg/L	In the embryo assay, no mortality is observed at either dose, but malformations (curled claw, everted viscera) occur at the higher dose. In the estrogenic assay (MCF-7), weak estrogenic activity is detected. In the Ames test, mutagenicity index (MI) values are 1.4 and 1.3 at 150 μg/L and 1.5 μg/L, respectively, remaining below the genotoxic threshold (MI ≥ 2.0)	Developmental toxicity is observed at a high dose, weak estrogenic activity is detected, and no genotoxicity is observed within the tested concentrations	(50)	2021

#### 3.1.3 *In silico* toxicity

*In silico* studies, the theoretical toxicity and endocrine disruption properties of THY and its derivatives were evaluated. These analyses, which use advanced computational modeling, help predict potential adverse effects and interactions with biological systems without requiring *in vivo* or *in vitro* testing. Some studies conducted *in silico* evaluations, using software to model both general toxicity parameters and hormone receptor interactions. Their findings provided insights into THY's potential as a therapeutic compound, with specific attention to its drug-likeness, resistance-evading potential, and endocrine-disrupting properties (see Table 3).

**Table 3:** *In silico* toxicity and endocrine disruption potential of THY and its derivatives.

***In silico* methodology**	**Objective**	**Key findings**	**Conclusion**	**References**	**Year**
Analysis using Osiris DataWarrior^®^ software	To evaluate THY and derivatives toxicity	THY and its derivatives exhibit theoretical toxicity parameters similar to existing antibiotics. Derivatives show negative drug-likeness values, suggesting originality and potential to evade resistance mechanisms. THY is flagged as potentially mutagenic, tumorigenic, irritant, and likely to cause reproductive effects.	THY and derivatives present theoretical risks; however, derivative structures may retain efficacy against resistant strains due to their unique drug-likeness profiles.	(51)	2020
PreADMET	To predict the toxicological effects of THY and phytol isolated from *Moringa oleifera*	THY tests positive in the Ames test for mutagenicity in three strains. Phytol shows potential carcinogenicity in rats.	THY raises concerns regarding mutagenicity, while phytol may have carcinogenic effects. Further toxicological studies are necessary.	(52)	
Docking on interface for target system's platform	To assess THY endocrine disruption potential	THY shows low binding affinity to most endocrine receptors, except for antagonistic binding to the androgen receptor.	THY exhibits limited potential as an endocrine disruptor, though its effect on androgen receptors warrants further investigation.	(50)	2021

#### 3.1.4 Ecotoxicity

The ecotoxicity of THY has been evaluated through studies assessing its potential impact on non-target organisms, including insects, microbial communities, and bioindicator species. Research has focused on understanding its effects on insect populations involved in ecological processes like nutrient cycling, as well as its influence on soil and aquatic microbial communities, examining parameters such as biodegradation capacity and metabolic activity. Additionally, the toxicity of THY has been tested using standard bioindicators, including aquatic organisms, earthworms, and plants, to determine its environmental safety profile. These investigations aim to provide a comprehensive understanding of thymol's behavior in different ecosystems and its suitability as an alternative to conventional synthetic compounds. These findings, summarized in Table 4, supported THY's potential as a sustainable alternative to synthetic compounds.

**Table 4:** Ecotoxicological assessment of THY- and THY-containing blends on non-target insect species.

**Model species**	**Treatment**	**Concentrations tested**	**Results**	**Conclusion**	**References**	**Year**
Dung beetle (*Ateuchetus cicatricosus)*	THY-CAR blend (25–25%)	0.1, 1.0, 10.0, 100.0, 1,000.0 mg/kg; dung from cows fed 300 mg/day for 7 days	No ecotoxic effects are observed at any concentration, including 1,000 mg/kg, which is ~1,000 × field-relevant exposure	The THY-CAR blend appears ecologically safe, showing no toxicity even at concentrations far above expected field exposure levels, making it a viable alternative to traditional veterinary products like IVM	(53)	2023
*Vibrio fischeri*	THY	0.02, 0.2, 2, 20, and 200 mg/L	Acute toxicity is observed at low concentrations (LC_50_ = 1.71 mg/L)	THY poses a toxicity risk to aquatic bioindicators at low concentrations	(54)	2024
*Daphnia magna*		2, 4, 6, 8, and 12 mg/L	Moderate toxicity is observed (LC_50_ = 8.13 mg/L)	THY exhibits toxicity to *D. magna* at higher concentrations		
*Allium cepa*		0.03, 0.3, 3, 30, and 300 mg/L	Root growth inhibition is observed (LC_50_ = 4.05 mg/L)	THY demonstrates acute toxicity to plant bioindicators		
*Eisenia fétida*		0.1, 1, 5, 10, 25, and 50 mg/kg	Moderate toxicity is observed (LC_50_ = 7.68 mg/kg)	THY shows toxicity to *E. fetida* at moderate concentrations		
River microbial community		0.1, 10, 100, and 1,000 μg/mL	No significant growth inhibition is detected; reduced substrate utilization occurs at high concentrations (up to 100 mg/L)	River microbes exhibit resilience to THY exposure; minimal long-term effects are expected		
Soil microbial community			Decreased substrate utilization is observed, particularly for polymers and amines (EC50 120 h = 94.13 mg/L)	Soil microbes appear more sensitive to THY than aquatic microbes; potential for biodegradation exists		

### 3.2 THY pharmacokinetics

Table 5 summarizes the pharmacokinetic studies examining the ADME of THY across multiple animal species: rats, dairy cattle, broiler chickens, pigs, and rabbits, and *in silico* studies. Key pharmacokinetic parameters were investigated following different routes of administration, including intravenous injection, inhalation, oral, intramammary, and feed-supplemented applications.

**Table 5:** Pharmacokinetics of THY in rats, rabbits, broiler chickens, pigs, and dairy cattle.

**Species**	**Matrix**	**Methodology**	**Pharmacokinetic parameters**	**Key findings**	**References**	**Year**
Rats	Plasma	Inhalation of THY vapor at 500 ppm for 1 h in a sealed chamber	Half-life: ~3 h; AUC: 180 μg·h/mL	THY is rapidly absorbed, reaching peak plasma concentration within 30 min. Slightly longer half-life and higher AUC compared to intravenous administration	(49)	2019
	Plasma	Single intravenous injection of THY (10 mg/kg)	Half-life: ~2.5 h; AUC: 150 μg·h/mL	Rapid increase in plasma THY post-injection; quick distribution phase, followed by a decline in plasma levels	(55)	
Rabbits	Plasma	Feed. THY, 250 mg/kg, 21 days, 7 days of withdrawal	C_max_: 0.05 + 0.02 μg/L	Plasma THY concentration significantly correlates with intestinal wall THY concentration	(56)	2021
Broiler chickens	Plasma	Feed. THEO (THY:58%): 0.1, 0.2, 0.3, 1% w/w for 35 days	Peak plasma concentration: 412.2 ± 170.7 ng/mL (1% THEO), significantly higher than that observed in the other supplementation levels	Systemic absorption of THY results in significantly higher plasma levels at higher THEO concentrations	(57)	2013
	Plasma	Feed. THEO 0.01–0.1% w/w	Peak concentrations: 90 to 850 ng/mL	Higher plasma THY concentrations with increased THEO supplementation	(58)	2016
	Plasma	Feed. THEO 0.05% (248.97 μg/g of THY) and 0.1% (460.22 μg/g of THY) w/w	Higher concentration at 0.1% addition	Significant increase in plasma THY levels with 0.1% THEO compared to 0.05%	(59)	2019
Pigs	Jejune (everted segments)	THY and THY-β-D-glucopyranoside: 100 μM; 120 min	THY peak serosal concentration: 3.2 ± 0.4 μM at 60 min THY-β-D-glucopyranoside peak serosal concentration: 1.8 ± 0.3 μM at 120 min	THY shows rapid absorption, outperforming THY-β-D-glucopyranoside. Its smaller molecular size and lipophilicity enhance bioavailability	(60)	2013
	Plasma	Feed. Free THY: 0.05 g/kg. THY with β-cyclodextrin encapsulation: 0.03 g/kg	Free THY parameters: Tmax 1.3 h, C_max_ 3.6 μg/ml, AUC0-10 h 17.3 μg h/ml. Enhanced absorption with β-cyclodextrin. Encapsulation prolongs THY's half-life, reducing daily administrations. Bioavailability remains unchanged compared to free THY	Faster absorption with β-cyclodextrin. Encapsulation prolongs THY's half-life, reducing administration frequency. Bioavailability remains unchanged	(61)	2014
	Plasma	Feed. THY with methylcellulose microencapsulation: 0.03 g/kg THY with hydroxylpropyl methylcellulose phthalate microencapsulation: 0.035 g/kg	Methylcellulose: Tmax: 0.5 h; C_max_: ~5 × higher than free THY; Hydroxylpropyl methylcellulose: similar pharmacokinetics to free THY	Enhances absorption with methylcellulose. Hydroxylpropyl methylcellulose reduces THY half-life but maintains similar absorption profiles	(62)	
Dairy cattle	Plasma	Intramammary Low dose: 240 mg of THY/quarter High dose: 480 mg of THY/quarter	Low dose: half-life:2.101 h; Tmax: 0.5 h; C_max_: 0.035 μg/mL; AUC: 0.137 μg·h/mL High dose: half-life:1.721 h; Tmax: 0.5 h; C_max_: 0.092 μg/mL; AUC: 0.252 μg·h/mL	Systemic absorption observed. Higher AUC in the high-dose group; no significant drug accumulation.	(63)	2016
	Plasma	Intramammary 120 mg THY/quarter/12 h; double dose also tested	Half-life: ~1.6 h	Higher AUC observed at higher doses, but the increase is not proportional	(64)	2017
Sheep	Plasma	Oral administration of THY (150 mg/kg) alone and co-administered with albendazole (ABZ; 5 mg/kg) to lambs	*1st administration*: C_max_ (ng/mL): 217 ± 80.4 aA, Tmax (h): 1.96 ± 1.83, T½ el (h): 5.47 ± 1.62, AUC0-t (ng h/mL): 1,351 ± 521 *2nd administration:* C_max_ (ng/mL): 1,772 ± 1,079, Tmax (h): 1.03 ± 0.08, T½ el (h): 16.0 ± 10.6, AUC0-t (ng h/mL): 7,650 ± 6,397 *1st administration* (with ABZ): C_max_ (ng/mL): 822 ± 457, Tmax (h): 1.00 ± 0.00, T½ el (h): 4.23 ± 1.26, AUC0-t (ng h/mL): 3,375 ± 1,910. *2nd administration* (with ABZ): C_max_ (ng/mL): 1,877 ± 1,120, Tmax (h): 1.00 ± 0.00, T½ el (h): 9.02 ± 3.29, AUC0-t (ng h/mL): 6,906 ± 3,481.	Coadministration with ABZ increases C_max_ and AUC compared to THY alone. The T½ el is significantly reduced after the second administration of THY with ABZ. Tmax remains consistent across both treatments. The second dose of THY alone shows higher C_max_ and AUC compared to the first dose, indicating increased absorption or bioavailability	(65)	2020
*In silico*	-	-	ADME	THY demonstrates good absorption, ability to cross the blood-brain barrier, and inhibition of CYP3A4 and P-glycoprotein, suggesting potential for drug interactions	(52)	2020

### 3.3 THY residue dynamics in dairy cattle, pigs, broiler chickens and quails

Table 6 presents a summary of THY residue studies across dairy cattle, broiler chickens, quails, and pigs, highlighting the absorption, distribution, and persistence of THY in different tissues.

**Table 6:** Summary of THY residue dynamics in food-producing animals.

**Species**	**THY source/ administration**	**Sample matrices**	**Findings**	**Key conclusions**	**References**	**Year**
Dairy cattle	Intramammary	Milk, liver, kidney	Residues are detected in milk up to 36 h, liver up to 96 h	THY persists longer in liver, a key organ for metabolism	(63)	2016
	Intramammary	Liver	Prolonged clearance is observed in liver; liver is recommended as a primary testing site	Liver acts as a principal residue storage site	(64)	2017
	Feed. THEO	Milk	The recommended milk withdrawal period is 72 h	Ensures consumer safety by reducing residue exposure	(66)	2022
	Feed. THEO (21 days)	Milk	Minimal THY levels are detected	Minimal residue risk in milk	(67)	2023
	Feed. THY-CAR blend	Feces	Low residual levels are found in feces, suggesting minimal environmental impact	Low risk of environmental contamination post-supplementation	(53)	
Pigs	Encapsulated and free THY (oral)	Liver, lungs, kidneys, gastrointestinal contents	Low residues are found in organs; higher concentrations in gastrointestinal contents	Limited systemic deposition; effective metabolic processing	(61)	2014
Broiler chickens	Feed. THEO	Duodenum, Jejunum, Liver	Highest concentrations are observed in duodenum and jejunum; low levels are found in liver	Efficient metabolism and clearance in tissues	(57)	2013
	Feed. THY	Duodenum	Biotransformation occurs into sulfate and glucuronide metabolites; highest levels are found in duodenum	Supports safe THY use due to efficient metabolism	(68)	2017
	Feed. THY	Muscle	Only trace residues are detected in muscle	Low risk of residue in edible muscle tissue	(69)	
	Feed. THY	Muscle	Trace concentrations are detected after extended feeding	Minimal tissue residue risk	(59)	2019
	Feed. THEO	Duodenum to Caecum, Plasma	A concentration gradient is observed from duodenum to caecum; plasma levels correlate with gut concentration	THY is absorbed systemically with minimal tissue residues	(70)	
Quails	Feed. THY	Egg yolk, Droppings	Dose-dependent increases are observed in egg yolk THY levels; levels decrease upon withdrawal	THY is deposited in eggs and decreases after withdrawal	(71)	2019

### 3.4 THY interactions with other compounds

Studies on the interactions between THY and other compounds in animal models have remained limited, particularly in the context of its pharmacokinetic and pharmacodynamic effects. The available studies have suggested that THY's interaction at the level of drug-metabolizing enzymes and related reactions, such as oxidation, reduction, and conjugation, may influence the efficacy and metabolic profile of co-administered compounds. However, understanding these interactions has remained an emerging area, with few detailed assessments, especially concerning THY's impact on ADME in production animals. In one of the few studies addressing this topic (29), the chemical-drug interaction between THY (150 mg/kg, administered two times every 24 h) and the antiparasitic agent ABZ (5 mg/kg) in lambs with resistant gastrointestinal nematodes was investigated. The study has found minimal metabolism of THY within the ruminal content and a low degree of association with the particulate phase. Notably, the pharmacokinetics of ABZ sulphoxide were unaffected by the presence of THY. However, co-administration has led to a significant reduction in the C_max_ and AUC of ABZ sulfone compared to ABZ-only treatment. Additionally, the presence of THY has not enhanced ABZ's efficacy but notably inhibited the ruminal sulphoreduction and hepatic sulphonation of ABZ sulphoxide.

### 3.5 THY pharmacodynamics

THY exerts a broad spectrum of biological activities through multiple mechanisms of action. It disrupts cell membranes by integrating into the lipid bilayer, which increases membrane permeability and causes the leakage of essential intracellular components, ultimately leading to cell death (72). This membrane-disrupting effect is central to its antibacterial properties, as THY also inhibits quorum sensing in bacteria, reducing bacterial communication, biofilm formation, and virulence. Furthermore, THY damages bacterial DNA and RNA, impairing replication and transcription, and it inhibits key metabolic enzymes, such as adenosine triphosphatases (ATPases) and glycolytic enzymes, vital for bacterial energy production. These combined actions contribute to THY's effectiveness as an antibacterial agent. In addition to its antibacterial effects, THY demonstrates potent antiviral activity by destabilizing viral envelopes or capsids, inhibiting viral replication, and interfering with viral entry into host cells. It also exhibits antileishmanial activity through its interaction with membrane sterols, disrupting membrane integrity and fluidity, leading to oxidative stress and cellular damage in parasites. Furthermore, in parasitic cells, THY triggers chromatin condensation, DNA fragmentation, and mitochondrial dysfunction, which are hallmark features of apoptosis-like cell death. THY's antifungal effects are evident through its disruption of fungal cell membranes by altering fatty acid metabolism, reducing ergosterol content, and increasing reactive oxygen species (ROS), ultimately causing oxidative stress and fungal cell death.

THY's ability to induce apoptosis is another key feature of its biological activity. It induces apoptosis in cancer cells *via* mitochondrial depolarization, activation of the Bax protein, caspase activation, and increased ROS levels (36), all leading to DNA damage and cell death. This makes THY a promising candidate for anticancer applications (73). In addition to its antimicrobial and anticancer properties, THY also demonstrates significant anti-inflammatory effects. These effects are attributed to its ability to inhibit the recruitment of cytokines and chemokines and modulate NF-κB signaling. This results in the downregulation of pro-inflammatory cytokines (TNF-α, IL-1β, IL-6), cyclooxygenase-2 (COX-2), and inducible nitric oxide synthase (iNOS), thereby reducing nitric oxide production (74). THY also chelates metal ions, such as iron and magnesium, which disrupt enzymatic activity and stabilize biofilms, further contributing to its antimicrobial and anti-inflammatory actions (73). THY's antioxidant properties also play a vital role in protecting cells from oxidative stress. By scavenging free radicals and enhancing the activity of endogenous antioxidant enzymes, such as superoxide dismutase (SOD), glutathione (GSH) peroxidase (GPx), and catalase (CAT), THY supports cellular defense mechanisms and reduces cellular damage caused by oxidative stress. These antioxidant effects contribute to THY's protective role against various diseases, including inflammation and cancer (73, 74). Moreover, THY exhibits antihyperlipidemic effects by increasing high-density lipoprotein (HDL) cholesterol levels while decreasing low-density lipoprotein (LDL) cholesterol in circulation. These actions, together with THY's ability to stabilize cellular membranes and maintain ionic homeostasis, highlight its potential in managing lipid imbalances (74). In addition to its biological effects, THY also exerts anesthetic properties mediated through gamma-aminobutyric acid type A (GABA_A) receptors, though these effects are independent of benzodiazepine binding sites (75). THY also modulates intracellular calcium flux by interacting with calcium channels like TRPA1, which influences cellular signaling and physiological responses, including mast cell degranulation (73). Additionally, at low doses THY acts as an agonist of α1-, α2-, and β-adrenergic receptors; however, at higher doses, it behaves as an antagonist, thus demonstrating its efficacy in reducing ileal contractions (76) and enhancing the absorptive capacity of the intestinal mucosa as a dietary supplement (16). These diverse mechanisms of action underscore THY's potential as a natural, multifunctional agent for veterinary and therapeutic applications.

### 3.6 THY pharmacological effects

#### 3.6.1 Antibacterial properties of THY and THEO

THY is widely studied for its potent antibacterial effects. Research indicates that THY disrupts bacterial cell membranes, leading to the leakage of cellular contents and eventual bacterial death. Studies on both Gram-positive and Gram-negative bacteria show that THY is effective across a range of bacterial strains, significantly reducing colony-forming units (CFUs) in a dose-dependent way. Furthermore, the structure of THY, with its phenolic groups, plays a critical role in its antibacterial activity by enhancing its hydrophobic interactions with bacterial cell membranes. Table 7 details the source of THY, the pathogenic strains tested, methods employed to determine efficacy, and minimum inhibitory concentrations (MIC) or minimum bactericidal/fungicidal concentrations (MBC/MFC) reported in each study. Additional relevant observations about THY antimicrobial mechanisms, resistance potential, and synergistic effects are noted, thereby offering insights into THY's potential applications and limitations as an alternative antimicrobial agent.

**Table 7:** Summary of studies on the antimicrobial efficacy of THY and THEO against various pathogens.

**Pathogen(s)**	**THY source**	**Method**	**MIC**	**MBC/MFC**	**Observations**	**References**	**Year**
*Aeromonas salmonicida subsp. masoucida*, *A. salmonicida subsp. salmonicida*, *A. hydrophila*, *Edwardsiella tarda*, *Vibrio vulnificus*, *V. parahaemolyticus*, *V. anguillarum*	Pure THY	Disk diffusion	0.01–0.32 mg/mL	Not reported	Inhibits growth, including strains resistant to amoxicillin and lincomycin	(77)	2012
*Streptococcus mutans*, *St. salivarius*, *St. sanguinis*, *St. pyogenes*, *Enterecoccus feacalis*, *Pseudomonas aeruginosa*, *Lactobacillus acidophilus*, *Staphylococcus aureus*	THEO from three different *Thymus* species (38.5%, 49.10% and 56.02% THY)	Broth microdilution	2.5–160 g/mL	5–320 μg/mL	THEO from *T. serpyllum* shows the strongest activity	(32)	2013
*St. pyogenes*	THEO (43.6% THY)	Not specified	Not reported	Not reported	Significant antibacterial activity against *S. pyogenes*	(78)	
*E. coli* O157	Pure THY	Cell culture	Not reported	Not reported	Reduces invasion and expression of colonization genes in *E. coli* O157	(27)	2014
*Pseudomonas spp*.	THEO (77.5% THY)	Not specified	0.4 mg/mL	Not reported	High sensitivity in all evaluated strains	(79)	
*E. coli*, *S. aureus*, *P. aeruginosa*, *K. pneumoniae*	THY-enriched hydrogel	Disk diffusion	Not reported	Not reported	Significant activity against Gram-positive and Gram-negative bacteria	(46)	2018
*Escherichia coli*, *Salmonella typhimurium*, *S. aureus*	THEO (35.4% THY)	Well diffusion	Not reported	Not reported	Particularly effective against Gram-positive bacteria at all tested doses	(80)	2019
*E. coli*	Pure THY	Incremental subculture	Not reported	Not reported	Development of tolerance in *E. coli* with morphological and genetic changes observed	(81)	
*S. pseudintermedius*, *P. aeruginosa*, *M. pachydermatis*	THEO and OEO (THY and CAR)	Broth microdilution	0.02–0.25% (200–2,292 μg/mL)	2 dilutions higher	Superior antimicrobial activity of THY and CAR over THEO and OEO	(82)	
*S. aureus*	THY and derivatives	Broth microdilution	Not reported	Not reported	THY derivatives are effective	(51)	2020
*E. coli*, *S. entérica*, *S. aureus*	Oregano EO (OEO)	Broth microdilution	Not reported	Not reported	Inhibits *E. coli* and *S. aureus* growth, reduces microbial load in insect vectors	(83)	
*E. coli*	Pure THY	Incremental subculture	Not reported	Not reported	No resistance was induced in *E. coli* compared to amoxicillin	(84)	
*E. coli*, *S. enterica*	THY (hydrolytic derivative TBG)	Not specified	Not reported	Not reported	No significant effect of THY on antibiotic susceptibility	(85)	2021
*Enterococcus faecalis*, *St. canis*, *Proteus mirabilis*, *S. pseudintermedius*, *P. aeruginosa*	THY, OEO	Not specified	18.8–9,600 ppm	37.5–9,600 ppm	All compounds show antimicrobial activity against isolates	(86)	
*S. aureus*, *E. coli*, *St. uberis*	Pharmaceutical formulation (25% THY)	Broth microdilution	22.72–45.4 mg/mL	45.4–90.09 mg/mL	Strong antimicrobial activity against common mastitis pathogens	(66)	2022
*E. coli*, *T. pyogenes*	THEO, OEO and CIN	Not specified	>32,768 mg/mL	>32,768 mg/mL	Synergistic effects were observed with combined EOs against *T. pyogenes* and most *E. coli* strains	(5)	2023
*Prototheca bovis*	THY	Broth microdilution	0.06 to 0.25%		THY appears effective against *Prototheca* species, and may be useful for environmental disinfection in barn	(87)	2024

#### 3.6.2 Antiviral effects of THY and THEO

THY and THEO exhibit notable antiviral activity against a variety of viruses. Table 8 summarizes studies investigating the antiviral properties of THY and THEO's against different viral pathogens. Key findings, such as the inhibitory concentration (IC_50_), viral titer reductions, and the specific methods employed to assess antiviral activity, are detailed for each study.

**Table 8:** Summary of antiviral activity of THY and THEO against various viruses.

**Virus**	**THY source**	**Method**	**IC_50_/Effective concentrations**	**Results**	**Observations**	**References**	**Year**
*Herpes simplex virus type 1*	Pure THY	Virion inactivation	IC_50_: 7 μM	90% inactivation within 1 h	Electron microscopy shows that the hydrophilic group on the benzene ring is critical for antiviral action; minor effect of aliphatic side chains	(35)	2012
*Norovirus surrogates*, *Feline calicivirus, Murine norovirus, Hepatitis A*	Pure THY (0, 0.5, 1, 2%)	Dose-dependent titration	0.5% and 1% (undetectable FCV titers)/1–2% for Murine norovirus	FCV titer undetectable at 0.5%, 1%; Murine norovirus reduced by 1.66–2.45 log; no effect on Hepatitis A virus	Demonstrates dose-dependent effect for FCV and MNV, but no efficacy against Hepatitis A virus	(38)	2015
*Feline coronavirus (FCoV-II)*	THEO (47% THY)	Plaque reduction, quantitative PCR	27 μg/ml and 270 μg/ml	Reduction of 2 log_10_ TCID 50/50 μl at 27 μg/ml; virucidal activity up to 3.25 log_10_ at 270 μg/ml after 1 h	Significant reduction in FCoV-II titer, indicating strong antiviral and virucidal activity	(88)	2021
*Feline calicivirus (FCV)*	THEO (47% THY)	Cytopathic effect titration	194.98 μg/mL (max. non-cytotoxic) to 19,498.40 μg/mL (100-fold over threshold)	No significant reduction in FCV viral titers at any concentration tested	THEO shows no virucidal effect on FCV despite THY content	(39)	2024

#### 3.6.3 Antifungal effect of THY and THEO

The antifungal properties of THY and THEO have been documented against a range of fungal pathogens. Studies report effective inhibition of mycelial growth and spore germination. Table 9 presents data on minimum inhibitory concentrations (MICs) and minimum fungicidal concentrations (MFCs) against various fungal strains, as well as outcomes in studies involving alternative delivery methods, such as vapor-phase application, and *in vivo* testing on fish survival.

**Table 9:** Antifungal activity of THY and THEO against various fungal strains.

**Fungus/Strain tested**	**Treatment and concentration**	**MIC (μg/mL)**	**MFC (μg/mL)**	**Findings**	**References**	**Year**
*Rhizopus oryzae*	THY and THEO (10 μL)	128–512	512–1,024	Inhibits mycelial growth and spore germination	(89)	2012
*Candida spp*.	THEO from 3 *Thymus s*pecies (THY: 38.5%, 49.10%, and 56.02%)	1–40	5–80	*T. serpyllum* shows strongest activity; is effective across strains	(32)	2013
*Pythium insidiosum*	THY with Itraconazole and Clarithromycin	n/a	n/a	Combination therapy enhances effectiveness (up to 96% inhibition)	(9)	2015
*Saprolegnia spp*.	THEO supplemented diet (0.5–2%)	n/a	n/a	100% fish (*Cyprinus carpio*) survival; significantly increases survival vs. control	(90)	2017
*Aspergillus flavus*	Liquid (20 μg/mL) andVapor (400 μg/mL)	n/a	n/a	Inhibits fungal growth and reduces aflatoxin B1 production; gene expression suppressed in vapor	(10)	2019
*Dermatomycetes* and *Mold Fungi*	THEO (10 μg/μL)	0.5–2.5	0.5–5	Shows significant fungicidal effect; inhibits skin-infecting fungi	(91)	2020
*M. pachydermatis*	THEO (THY = 19%)	n/a	n/a	Inhibites all isolates from canine ears	(92)	

#### 3.6.4 Antiparasitic effect of THY and THEOs

##### 3.6.4.1 Ectoparasites

THY and THEOs are effective against a range of ectoparasites, including tick species like *Rhipicephalus microplus* and *Ixodes Ricinus* and poultry mites. Additionally, THY is proven effective as a repellent and insecticide for pests such as the lesser mealworm (*Alphitobius diaperinus*) and houseflies (*Musca domestica*), supporting its role as a versatile natural antiparasitic.

##### 3.6.4.2 Endoparasites

THY shows strong antiparasitic activity against protozoans like *Eimeria spp*. and *Cryptosporidium spp*., where it disrupts oocyst walls, suggesting its potential as a disinfectant for poultry. For nematodes, particularly *Haemonchus contortus*, THY effectively inhibits egg hatching and larval motility, with near-complete suppression *in vitro*. THY also induces structural damage and mortality in *Echinococcus spp*. and *Mesocestoides corti* (cestodes) larvae. Studies on *Leishmania spp*. further highlight the THY's ability to reduce parasite loads and enhance host survival, indicating its promise in treating leishmaniasis. THY's interaction with drugs like ABZ suggests a potential role as a co-treatment, though its metabolic impact warrants further study.

Table 10 summarizes THY's antiparasitic effects across multiple studies, covering both ecto- and endoparasites, including flies, ticks, mites, and various parasite stages and species like coccidia, nematodes, cestodes, and *Leishmania*.

**Table 10:** Antiparasitic activity of THY and THEOs against various ectoparasites and endoparasites.

**Target parasite**	**Treatment and concentration**	**LC_50_ or Mortality rate (%)**	**Findings**	**References**	**Year**
**Ectoparasites**					
*Musca domestica* (fly)	THY (2 g/kg) in quail diet	n/a	Reduces fly emergence and oviposition; significant oviposition repellent effect	(4)	2014
*Culex pipiens* (mosquito)	THY-CAR blend; 4:1	LC_50_ larvae: 14 mg/L	Shows synergistic ovicidal and larvicidal effects; THY-CAR combinations are effective	(6)	2019
*Rhipicephalus microplus* (cattle tick)	*Lippia gracilis* EO (THY = 59.6%)	LC_50_: 0.84–1.02	Shows significant acaricidal effect; is effective on resistant and susceptible strains	(11)	2016
*Rhipicephalus annulatus* (cattle tick)	THY-IVM; 1:1.	LC_50_ of the combination is not reported	Increases mortality, but the combination index exceeds one for all isolates, indicating no synergism	(93)	2024
*Ixodes ricinus* (livestock and dog tick)	THY (0.25–5%)	>90% repellency, 100% larvicidal	Shows high toxic and repellent activity; has superior efficacy compared to permethrin	(94)	2017
*Rhipicephalus sanguineus* (dog tick)	THY-EUG microemulsion (5 mg/mL each)	>90% reduction in infestations	Shows long-lasting acaricidal effect in field conditions; is well-tolerated on treated animals	(12)	2021
*Dermanyssus gallinae* (red mite)	THY-CAR; 4:1 (0.5–2%)	Prolonged efficacy (14 days)	Shows highest efficacy at 4:1 ratio with 2% concentration; demonstrates prolonged residual acaricidal effect	(95)	2016
*Alphitobius diaperinus* (poultry mealworm)	OEO	LC_50_: 0.135 μL/cm^2^	Shows high contact toxicity; is effective against pests in poultry settings	(83)	2020
**Endoparasites**					
*Eimeria spp*.	THY (0–20 mg/mL)	LC50: 1.66 ± 0.44 mg/mL	Shows significant oocysticidal effect, disrupts oocyst wall and membrane integrity, leading to parasite death	(96)	2013
*Eimeria tenella*	*In vitro*: THY (7 ppm)	Not specified	Reduces the invasion of MDBK cells by *Eimeria tenella* sporozoites	(97)	2020
*Echinococcus granulosus*	THEO and OEO	Not specified	Inhibits larval hatching and motility, highlighting potential for livestock nematode control	(98)	2014
*Mesocestoides* *corti*	THY	Not specified	Shows concentration-dependent morphological changes; results in complete larval mortality	(99)	
*Haemonchus* *contortus*	*In vivo*: THEO (300, 150, 75 mg/kg bw, days 0, 6, 12). *In vitro*: THEO (50–0.097 mg/mL), THY (25–0.048 mg/mL)	*In vitro:* egg hatching: 96.4–100%, Larval development: 90.8–100%, Larval motility: 97–100%, Adult motility: 100% (8 h)	*In vivo*: ineffective at tested doses. *In vitro:* both oil and thymol were highly effective at all parasite stages, with results comparable to levamisole (20 mg/mL)	(100)	2016
	THY and THY acetate	Not specified	Inhibits larval hatching and motility, highlighting potential for livestock nematode control	(47)	2017
	THY (IC50: 0.13 mg/mL)	IC50: 0.13 mg/mL	THY shows potent ovicidal effect	(101)	
*Haemonchus spp.*, *Teladorsagia spp.*, *Trichostrongylus* *spp. (resistant strains)*	*In vivo**:*** ABZ (5 mg/kg), THY (150 mg/kg), two times, 0 and 24 h. Co-administered and alone. Egg counts: 0 and 14 days	THY efficacy: 0%	THY administered alone or combined with ABZ also fails to reduce eggs number in feces	(65)	2020
*Haemonchus spp.*, *Trichostrongylus spp.*, *Teladorsagiaspp., Chabertia spp*.	*In vitro:* THEO (50, 12.5, 3.125, 0.781, 0.195, 0.049 mg/mL) for 48 h (egg hatch test) *In vivo:* 100 mg/kg body weight of THEO as single dose	Anthelmintic effect: ~25%.	THEO shows significant reductions in nematode egg counts in the *in vivo* fecal egg count reduction test	(102)	2022
*Leishmania* *infantum*	Acetyl- and benzoyl-THY derivatives	EC50: 8.67 μg/mL	Achieves significant reductions in promastigote loads, indicating potential as a treatment for leishmaniasis	(3)	2014
	THY	Not specified	Reduces parasite loads and improves survival in infected hamsters	(103)	2019
*Cryptosporidium baileyi Cryptosporidium galli*	*In vitro:* THY and THEO (0, 0.25, 0.33, 0.5, 1, 2 mg/mL)	THY: LC_50_ < 0.4 mg/mL	Both show significant oocysticidal activity. Higher concentrations and longer exposure times increased efficacy. Structural damage to oocysts is observed. Effective as natural disinfectants for controlling *Cryptosporidium* infections in poultry	(104)	2019

#### 3.6.5 Antiinflammatory effects of THY

Data reported in Table 11 highlight THY's anti-inflammatory effects across a variety of animal models and *in vitro* systems, demonstrating its potential to modulate inflammation through multiple pathways. THY's anti-inflammatory properties are evident in rodent models, avian species, and cellular assays, with studies reporting reductions in edema, leukocyte infiltration, and pro-inflammatory cytokine expression. The studies utilized diverse methods, including THY alone or in combination with CAR, administered through various doses and routes. Key indicators of inflammation, such as cytokine levels (e.g., TNF-α, IL-1β), myeloperoxidase (MPO) activity, and oxidative stress markers, were measured to assess THY's effects.

**Table 11:** Summary of studies evaluating the anti-inflammatory effects of THY in veterinary species.

**Model/ system**	**Condition**	**THY doses/ administration**	**Key measurements**	**Main findings**	**References**	**Year**
Rat and mouse models	Ear Edema and Pleurisy	10, 100, 200, 400 mg/kg	Edema volume, inflammatory cell migration, chemotaxis	Inhibits edema in pleurisy model but shows no effect on cell migration; enhances chemotaxis	(43)	2012
	Paw Edema and Peritonitis	10, 30, 100 mg/kg, i.p.	MPO activity, total leukocyte count, histology	Reduces paw edema and leukocyte infiltration; shows dose-dependent reduction in MPO activity and cell count	(105)	
	Hepatic damage model: hydrocortisone-induced inflammation	3 mg/kg (oral)	TNF-α levels in serum and hepatic tissue	Decreases TNF-α; hypothesizes inhibition of TNF-α gene expression	(106)	2015
	Elastase-induced pulmonary emphysema	30-min post-elastase, repeated doses on days 7, 14, 28	IL-1, IL-6, IL-8, IL-17, MMP-9, NF-κB, nitric oxide	Reduces emphysema, inflammatory markers, and oxidative stress	(107)	2016
	LPS-induced endometritis	THY + LPS	TNF-α, IL-1β, MPO, NF-κB, ROS signaling	Alleviates MPO activity, TNF-α, IL-1β; suppresses NF-κB *via* TLR4, ROS	(41)	2017
	Induced immunomodulation	10, 20, 40 mg/kg for 30 days	NTPDase, ADA enzyme activities	Decreases NTPDase activity; increases ADA activity at higher doses	(48)	2018
	LPS-induced acute lung injury	20, 40, 80 mg/kg, i.p.	TNF-α, IL-6, IL-1β, MPO activity, NF-κB, Nrf2, HO-1	Reduced inflammatory cytokines, MPO activity, NF-κB signaling; increased Nrf2, HO-1	(108)	
	Drug-induced gastric ulcers	75, 100, 250, 500 mg/kg, oral	Liver enzymes, TNF-α, eNOS, caspase-3, TOS/TAC	Reduces TNF-α, oxidative stress markers, and caspase-3 activation; optimal dose at 250 mg/kg	(109)	
	Acetic acid-induced colitis	THY: 10, 30, 100 mg/kg per day (oral)	Macroscopic and histopathologic investigations, MPO and TNF-α expression (IHC), pNF-κBp65 protein expression (Western blot)	Reduces mucosal and histological damage, inhibits MPO and TNF-α production, and decreases pNF-κBp65 expression, suggesting anti-inflammatory effects via NF-κB pathway inhibition	(110)	2019
Weaned piglets	Healthy post-weaning piglets	EO's blend (18% THY, 0.01%)	IL-6, TNF-α	Decreases IL-6, higher TNF-α	(18)	2012
	Post-weaning inflammation	1:1 THY-CAR blend, 100 mg/kg	TNF-α mRNA expression in jejunum	Decreases TNF-α expression in jejunal mucosa	(15)	2017
Broiler chickens	*C. perfringens* challenge	25–25% blend with CAR	TLR2, IL-1β, TNF-α gene expression	Downregulates TLR2 and TNF-α; reduces inflammatory response	(111)	2016
Rabbit	High-fat diet-induced inflammation	3 mg/kg/day, 6 mg/kg/day	VCAM-1, MCP, IL-1β, IL-6, TNF-α, C-reactive protein	Reduces inflammatory markers, VCAM-1, MCP, IL-1β, IL-6, TNF-α; reduced CRP	(112)	2016
*In vitro*	LPS-induced Inflammation	10, 20, 40 μg/mL	TNF-α, IL-6, IL-1β, COX-2, iNOS, NF-κB, MAPK pathway	Reduces cytokines, COX-2, iNOS; blocks NF-κB and MAPK activation	(24)	2013
	Th1/Th2 cytokine balance	0.05, 0.5, 5 μM	Cytokines (IL-2, IFN-γ, IL-4, IL-5, IL-10)	Decreases IL-2, increases IL-10/IL-2 ratio; suggests shift toward Th2 balance	(40)	
	LPS-induced inflammation	20, 40 μM (cells), 25, 100 mg/kg (mice)	TNF-α, IL-1β levels	Inhibites TNF-α, IL-1β in cells; reduces TNF-α in mice, IL-1β effect less significant	(26)	2018
	LPS-induced inflammation	THY: 10 ppm and THEO: 20 ppm (6-h exposure)	FD4 paracellular permeability (PCP), gene expression (IL1β, IL6, IL8, TNF-α, IFN-γ, defensin, and cathelicidin-2)	THY and THEO reduce FD4 PCP (30 and 40%, respectively), decrease pro-inflammatory markers and enhance defensin and cathelicidin-2 expression	(113)	2023

#### 3.6.6 Antioxidant effects of THY across animal models and *in vitro* systems

Table 12 provides a summary of studies that evaluated the antioxidant effects of THY across multiple species and models. THY consistently enhances antioxidant activity through reductions in lipid peroxidation markers, such as malondialdehyde (MDA), and increases in enzymatic antioxidants like SOD and GPx. These effects are observed across aquatic, mammalian, and avian species, as well as in *in vitro* studies, highlighting THY's broad-spectrum potential to mitigate oxidative stress.

**Table 12:** Antioxidant effects of THY across multiple species and models.

**Species/ model**	**THY Dosage/ formulation**	**Key measurements**	**Findings**	**References**	**Year**
**Aquatic species**					
Nile tilapia	1–2 mL/kg feed	MDA, GSH reductase in muscle/liver, catalase	Reduces MDA, enhances antioxidant enzyme activity	(2)	2018
	Thyme powder (THP; 2%) with insecticide exposure	MDA in *O. niloticus*	Reduces MDA levels compared to control; mitigates insecticide-induced oxidative damage	(13)	2020
Rainbow trout	Phytogenic additive (6 g/kg of THY)	MDA, GSH-based enzymes, catalase	Reduces MDA, increases GSH activity, increases catalase	(114)	2012
	0, 500, 1,000, 1,500 mg/kg THEO	SOD, GPx, MDA	Increases liver SOD and GPx, reduces MDA	(115)	2015
	THEO	ALT, AST, cortisol, glucose, CAT, GPx	THY suppresses oxytetracycline-induced enzyme changes; increases gut CAT and GPx activities	(116)	2018
Common carp	THY (short/long exposure)	MDA, oxidative stress markers	Lower MDA, less stress-induced oxidative markers with THY	(117)	2018
**Mammalian models**					
Mice	THY (60 mg/kg in DSS model)	MPO, MDA, GSH, SOD	Loweres MPO and MDA, increases GSH and SOD in colonic tissues, indicating antioxidant effect in DSS colitis model	(45)	2018
	THY (0, 20, 40, 80 mg/kg)	Lung MDA	THY dose-dependently reduces LPS-induced MDA in lung tissue	(108)	2018
Rats	THEO	Lipid peroxidation, SOD, GSH	Reduces lipid peroxidation and restores SOD and GSH levels in *T. vitulorum*-infected rats	(118)	2013
	THEO (5.6% THY) and olive EOs (1.5 g/kg bw)	SOD, GPx, CAT, non-enzymatic antioxidant capacity	Increases catalase, reduces SOD and GPx, modulates plasma antioxidant capacity; enhances bioavailability of olive phenolics in combination	(119)	2014
	THY oral administration	TAC, TOC, TBARS, GSH	Reverses hydrocortisone-induced oxidative stress markers, elevates TAC, and decreases TOC	(106)	2015
Rabbits	THY (3–6 mg/kg/day)	MDA, serum TAC	Lowers MDA and enhances TAC in the serum of high-fat diet animals	(112)	2016
	THY (250 mg/kg feed)	MDA, GPx, LDH	Reduces MDA and GPx in blood; decreases LDH in muscle tissue	(120)	2020
Pigs	EOs blend (18% THY, 0.01%)	Plasma TAC	Increases TAC	(18)	2012
	EOs blend (13.5% THY, 4.5% CIN)	Plasma antioxidant capacity	Increases plasma antioxidant capacity	(121)	2014
	THY-CAR blend (1:1, 100 mg/kg)	SOD, GPx, TBARS in jejunum	Elevates SOD, GPx, and reduces TBARS in jejunum in supplemented group	(15)	2017
	THY (0.01% in diet)	SDH, LDH, drip loss	Reduces oxidative markers, enhances SDH activity, and improves muscle quality	(122)	2020
	EOs blend (10% THY, 10% CIN)	Hepatic SOD, GSH	Higher hepatic SOD activity; no reduction in GSH, suggesting stimulated antioxidant defense	(123)	2023
Dairy cows	THEO (THY ~59%)	NF-κB binding energy	Demonstrates potential against oxidative stress, with binding energy of THY to NF-κB indicating suitability for oxidative stress management	(124)	2023
**Poultry**					
Broiler chickens	THY-CAR blend (0, 60, 100, and 200 mg/kg)	SOD, GPx, MDA	Increases SOD, GPx; decreases MDA in liver, serum, and thigh muscle	(125)	2013
	THY (200 mg/kg)	Plasma MDA	Significantly reduces MDA in plasma	(22)	2014
	*Nigella* seed EO (0, 0.5, 1, and 1.5 g/kg)	SOD, GSH, GPx	Greater SOD and GSH with 1.5 g/kg; increases GPx in antibiotic and 1 g/kg groups	(21)	2015
	THY (250 mg/kg feed)	Serum GPx	Elevates GPx in heat-stressed chickens	(126)	2016
	THEO (0, 0.05, and 0.1%)	SOD, MDA	Elevates SOD and reduces MDA levels in plasma with THEO supplementation	(59)	2019
	EOs blend (0–400 mg/kg)	SOD, MDA, GPx, total antioxidant capacity	SOD increases in higher doses (200–400 mg/kg); no significant effect on MDA, GPx, and total antioxidant capacity	(19)	2021
Turkey	THY (0, 30 mg/kg) or EO blend	Lipid oxidative stability, GSH enzymes	Improves lipid oxidative stability and increases GSH-based enzyme activity	(127)	2014
Laying hens	THP (0–9 g/kg)	SOD, GSH, MDA	Increases SOD, GSH; reduces MDA	(128)	2015
THP (0.9%)	SOD, MDA	Increases SOD, decreases MDA levels	(1)	2017
***In vitro*** **and** ***ex vivo*** **studies**					
Porcine brain tissue	THEO from three different *Thymus* species (THY: 38.5%, 49.10%, and 56.02%)	Radical scavenging, thiobarbituric acid assay	Highest radical scavenging in *T. serpyllum*, followed by *T. algeriensis*, and *T. vulgaris* Eos	(32)	2013
RAW264.7 macrophages	THY (10, 20, and 40 μg/mL)	ROS	Dose-dependent inhibition of LPS-induced ROS production	(41)	2017
IPC-J2 cells	THY pretreatment (0, 50 μM)	ROS	Inhibites LPS-induced ROS production	(25)	2019
Bovine ovarian tissue	THY (400, 800, 1,600, and 3,200 μg/mL)	mRNA for SOD1, CAT, GPX1, PRDX6	Increases CAT activity at 800 μg/mL; reduces mRNA expression of SOD1, CAT, PRDX6 at 400–800 μg/mL	(129)	2024

#### 3.6.7 Hemato-biochemical effects of THY, THEO, and other EOs containing THY

The hemato-biochemical effects of THY and THEO have been widely studied across various animal models. Across species, THY and THEO consistently show beneficial effects. Table 13 summarizes findings from studies on their effects in multiple species, including mammals, poultry, fish, and other livestock animals.

**Table 13:** Effects of THY, THEO, and other THY-containing EOs on hemato-biochemical indices in animal models.

**Species**	**THY/Thyme formulation**	**Dose**	**Observed effects**	**References**	**Year**
Rats	THEO	Not specified	Decrease in RBC, Hb, lymphocyte%, and MCHC; increase in WBC, monocytes, granulocytes, AST, ALT, urea, creatinine, albumin, and globulin; amelioration of Toxocara-induced changes	(118)	2013
	THY	30 mg/100 g bw	Reduces ALT and AST levels in hydrocortisone-induced damage animals	(106)	2015
Rabbits	THY	3–6 mg/kg	Reduces cholesterol, triacylglycerol, LDL; increases HDL with high-dose THY	(112)	2016
	THY	250 mg/kg feed, 21 days	Decreases ALP (*p* = 0.0183) and cholesterol (*p* = 0.0228); increases urea (*p* = 0.0079) upon withdrawal; decreases triglycerides in THY withdrawal group	(120)	2020
Weaned pigs	*Lippia origanoides*-*E. caryophyllata* EOs (47.5% THY)	67 mg/kg and 75 mg/kg blends	No changes in cortisol levels; increases plasma citrulline and disaccharidase activities in post-weaning stressed pigs	(20)	2021
Goats	THEO	0.5–2 ml/kg feed	No significant effect on ALT; reduces AST at 0.5 ml/kg; decreases ALP at 2 ml/kg after 60 days	(14)	2019
	EO's blend (10% THY, 10% CIN, others)	0, 100, 200, 400 mg/kg for 42 days	Reduces total protein and plasma protein at 100 mg/kg; significant hematological changes	(123)	2023
Dogs	THEO	Oral administration, 20% conc.	No significant changes in biochemical parameters except serum urea levels	(130)	2023
Broilers	THY-CAR	0, 60, 100, 200 mg/kg diet	Increases trypsin, lipase, and protease activities in 24-day-old birds; no effect in 42-day-old birds	(125)	2013
	THEO	0, 5, 7.5 g/kg diet	Lowers serum protein, albumin, cholesterol, and triglyceride at 7.5 g/kg	(131)	
	THEO	0.05–0.35 mg/kg	Increases serum proteins and globulins; decreases ALT, albumin-to-globulin ratio, and urea; improves HDL levels	(132)	2014
	*Nigella* seed EOs	0.5–1.5 g/kg	Decreases plasma total protein, albumin, ALT, and ALP; variable plasma urea and creatinine; reduces lymphocyte, monocyte, eosinophil, and heterophile counts	(21)	2015
	THY + BA	THY: 100–200; BA: 1,000–2,000 mg/kg	No effect on albumin, triglyceride, cholesterol, globulin, urea; increases lipase, lactase, and sucrose in jejunum	(133)	
	THP	0.9%	Significant effects on all serum constituents except total protein, albumin, and HDL	(1)	2017
	THEO	1–2 g/kg feed	Increases plasma protein and globulin; decreases albumin/globulin ratio, and AST; enhances WBC	(134)	
	THP	0, 2, 5, 8 g/kg	Increases WBC, lymphocyte count, IgG, NO; decreases cholesterol, LDL; increases HDL	(135)	
	THP	10–30 g/kg diet	Increases plasma total protein, hemoglobin, and MCH with high doses	(136)	2018
	THEO	3 g/kg diet	Improves HDL in aflatoxin- and ochratoxin-contaminated diets	(137)	2019
	THY and other THY-containing EOs	Not specified	Significant increase in eosinophils, lymphocytes, basophils, and monocytes % (*p* < 0.05) in all supplemented groups; higher WBC, total protein, albumin, lower cholesterol, and triglycerides	(138)	2020
	EOs blend with THY (main compound, 3.05%)	0, 50, 100, 200, 400 mg/kg	Increases ileal sucrase activity (*p* < 0.05); quadratic increase in sucrase activity with EOs levels	(19)	2021
	THEO, OEO and other THY-containing E0's (star anise, rosemary)	25 mg/kg, THY = 1.02 mg/kg	Increases trypsin, chymotrypsin, and elastase activities in EOs plus saponins group	(139)	
Laying hens	THP	0, 3, 6, 9 g/kg diet	Reduces triglycerides, LDL; lowers total cholesterol with 3 g/kg THP	(128)	2015
	THEO + peppermint EOs	100 mg/kg	Decreases serum cholesterol; increases eggshell thickness and Haugh unit in cold-stressed hens	(140)	2016
Quail	THEO + savory EO	200–400 ppm	Reduces triglycerides in both sexes; decreases cholesterol in males	(141)	2018
	THEO	400 ppm	Decreases triglycerides in male birds at high concentrations	(80)	2019
Ducks	THEO	50–100 mg/kg	No significant effect on serum protein, albumin, globulin, cholesterol, triglycerides, ALT, or AST	(142)	2019
Pigeons	THY	40 mg/kg bw for 15 days	Decreases ALT and AST in THY-treated infected birds; no significant changes in albumin, creatinine, or electrolyte levels	(143)	2020
Tilapia	THEO	1%	Enhances hematocrit, WBC, RBC, neutrophil, and monocyte counts	(144)	2014
Rainbow trout	THEO and fennel EO blend	10 ml·100 g^−1^ (1-week supplementation)	Enhance bactericidal activity, increase total protein, albumin, cholesterol, triglyceride, and bilirubin; decrease plasma glucose; increase K, Na, Ca, and Mg levels; decrease Cl levels	(145)	2013
	THY	2–2.5 g	Increases Hb, RBC, hematocrit, WBC at higher doses; enhances protease and lipase activities; no significant amylase changes	(146)	2022
Tambaqui	THEO (THY = 55.9%)	50 mg/L	Loweres glucose levels post-anesthesia; increases glucose upon recovery	(147)	2022
Common carp	THP	0.5–2%	Increases RBC, Hb, PCV, WBC; highest Hb, and PCV with 1.5% THP	(90)	2017
	THP (THY concentration not reported)	2% for 30 days	Modulates erythrogram indices altered by lambda-cyhalothrin insecticide exposure; no significant differences in Hb, RBC count, PCV%, and MCV without insecticide	(13)	2020
African catfish	THEO	500 ppm for 1 month	Reduces hepatorenal damage in thiamethoxam-intoxicated fish; partial restoration of serum hepatic enzymes and creatinine	(148)	2020

#### 3.6.8 THY and THEO supplementation effects on animal immunity

THY and THEO supplementation have shown positive impacts on immune function across multiple species, including poultry, fish, and mammals. Together, these findings underscore the potential of THY and THEO as dietary additives to support immune resilience in both livestock and aquaculture, though effects are dosage- and species-dependent. Table 14 summarizes findings from studies that evaluated immune responses to dietary THY or THEO supplementation in various animal species.

**Table 14:** Summary of immune responses to THY, THEO, and different blends of supplementation across animal species.

**Species**	**THY source and dosage**	**Duration**	**Key immune findings**	**References**	**Year**
Piglets	EO's blend (18% THY, 0.01%)	35 days	Increases lymphocyte proliferation	(18)	2012
	Encapsulated EO (THY + CIN)	Not specified	Increases lymphocyte transformation, IgA, IgM, C3, and C4 levels	(149)	
	EO blend (13.5% THY, 4.5% CIN, 0.025%)	28 days	Increases albumin, IgA, and IgG	(121)	2014
Blue foxes	THY (0, 100, 200, and 300 mg/kg)	30 days	100 mg/kg of THY increases IgA, IgG, and IgM	(150)	2024
Broilers	THEO (5 g/L) and THEO-CIN-turmeric (TUR) blend (5 g/L)	21 days	The blend improves immune response to Newcastle disease vaccine	(151)	2012
	THY-CAR (60–200 mg/kg)	Not specified	Enhances hypersensitivity response, IgG anti-sheep RBC titer, lower heterophil ratio	(125)	2013
	*Nigella* seed EO (0.5–1.5 g/kg)	45 days	Increases phagocyte index and antibody titer to Newcastle and infectious bursal disease viruses	(21)	2015
	THP (5 g/kg) and THEO-TUR blend (2.5 g/kg each)	Not specified	Increases Newcastle and Influenza virus antibody titers	(152)	2016
	THEO (0–2 g/kg)	Not specified	Increases antibody titers for infectious bursal disease virus at 2 g/kg	(134)	2017
	THP (10–30 g/kg)	28 days	Significantly increases antibody titer at 10 g/kg	(136)	2018
	THEO	76th day blood sample	Phagocytic activity increases significantly with THY group	(138)	2020
	EO's blend (THY 3.05%; 50–400 mg/kg)	42 days	Increases jejunal/ileal SIgA, serum IgG at 42 days with EOs supplementation	(19)	2021
Laying hens	THP (3–9 g/kg)	16 weeks	Increases IgG and IgA, particularly with 3 g/kg THP	(128)	2015
Rainbow trout	Phytogenic additive (6 g/kg of THY)	8 weeks	Increases lysozyme and total complement activity	(114)	2012
	THEO (THY 38.74%, 0.5–2 ml/kg)	2 months	Upregulates C3 and CD4 at 1–2 ml, higher lysozyme at 2 ml; IL-1β and lysozyme downregulates at 2 ml end of trial	(14)	2019
Nile tilapia	THY (1–2 ml/kg)	75 days, 3 × daily	Increases IgM, IgG, lysozyme	(2)	2018

#### 3.6.9 THY effects on disease resistance

Several studies examined the pharmaco-toxicological properties of THY, with a particular focus on its effects on disease resistance, immune modulation, and cellular response across various species, including poultry, livestock, fish, and rabbits. Researchers have explored the role of THY in combatting bacterial pathogens such as *Campylobacter spp., Salmonella spp., S. aureus, and St. iniae*, as well as its potential in reducing the impact of environmental stressors, including mycotoxin contamination, pesticide exposure, and heat-induced stress. Many studies underscored THY's ability to modulate immune responses, demonstrating increased antibody production, enhanced leukocyte activity, and decreased pathogen colonization in animal models, as previously mentioned. These findings offer promising insights into the therapeutic applications of THY as a natural alternative for enhancing disease resistance and mitigating toxicological impacts in veterinary medicine. Table 15 presents a comprehensive overview of studies evaluating the effects of THY and THEO on disease resistance in various animal species.

**Table 15:** Disease resistance effects of THY, THEO, and different blend supplementation in animal models.

**Species**	**THY/EO formulation**	**Dose**	**Observed effects**	**References**	**Year**
Rats	THY	30 mg/100 g bw	Protects liver from hydrocortisone-induced damage and restores normal liver architecture	(106)	2015
Pigs	THY	100–400 mg/kg	Reduces pancreatic cell damage in rats treated with THY and ketoprofen	(153)	2019
	THY-β-D-glucopyranoside (TBG)	6 or 18 mg/kg	Minimal effect on *Salmonella spp., E. coli*, or *Campylobacter spp*. concentrations in gut	(85)	2021
Bovine	THY	16–64 mg/mL	Inhibites *S. aureus* internalization in mammary epithelial cells; reduces NF-kB activation and nitric oxide production	(45)	2014
Rabbits	THEO	500 mg/kg, 5 days	Enhances IgG antibody response against *Eimeria stiedae*	(154)	2020
Broiler chickens	THY-CIN blend	50–100 mg/kg, 42 days	Reduces *Salmonella* spp. colonization in cecum at slaughter in birds given 50 mg/kg; highest colonization in control birds	(155)	2014
	THY and THY-CAR	0.25–2%	Reduces *Campylobacter spp*. counts in broiler chickens with 0.25% THY and 0.5% THY-CAR	(156)	
	THY-CAR	60, 120, or 240 mg/kg	Enhances immune response with higher Newcastle Disease Virus antibody titers in chickens given 120 and 240 mg/kg doses	(111)	2016
	Microencapsulated THY, CAR, sorbic acid	1 g/ton (initial phase), 2 g/ton (final phase)	Reduces *Salmonella spp*. serovar counts in liver and cecum; increases final body weight in treated chickens	(157)	2023
Japanese quail	THY	80 mg/quail per day	Increases globulins and decreased antibody titers; reduced stress-induced heterophil-to-lymphocyte ratio in heat-exposed quail	(158)	2019
Tilapia	THEO	1%	Enhances phagocytic activity and lower *St. iniae* mortality	(144)	2014
	THEO	0.5–2 ml/kg	At 0.5 ml/kg THEO diet, the highest survival rate is observed in rainbow trout after *Aeromonas hydrophila* challenge	(14)	2019
	THP	2%, 30 days	Modulates immune response; increases lysozyme, IgM, and complement levels in *O. niloticus* exposed to insecticide	(13)	2020
African catfish	THEO	500 ppm, 1 month	Reduces thiamethoxam-induced hepatorenal and immunotoxic damage; improves tissue structure in fish organs	(148)	2020
Rainbow trout	THY	1–2.5 g/kg	Increases lysozyme and phagocytic activity; reduces mortality from *St. iniae* challenge in high-dose THY group	(146)	2022
Shrimp	Microencapsulated THEO	0.5–1%	At 1% THEO diet, higher survival rates and reduced symptoms of White Spot Syndrome Virus (WSSV) infection are observed in shrimp	(159)	2018

#### 3.6.10 THY effects on productive performance parameters

The presented tables summarize studies investigating the impact of THY and THEO-based dietary supplements across various animal species, including poultry (Table 16), mammals (Table 17), and fish (Table 18). These studies are focused on evaluating the effects of different sources, concentrations, and durations of THY or THEO supplementation on productive parameters such as body weight, feed conversion ratio (FCR), growth rate, and other relevant metrics.

**Table 16:** Productive performance in birds fed with a diet supplemented with a THY source.

**THY source and concentration**	**Duration**	**Main findings on productive parameters**	**References**	**Year**
**Chickens**				
THEO (5 g/L); THEO-CIN-TUR blend (5 g/L in equal ratios)	1–21 days	Decreases live body weight and increases relative weight of organs; mix treatment decreases carcass weight compared to control	(151)	2012
THEO (thyme leaves; amount unspecified)	1–35 days	Normal performance; no losses; no performance enhancement noted under optimal conditions	(57)	2013
THY-CAR (0, 60, 100, and 200 mg/kg diet)	1–42 days	Reduces feed intake; highest body weight gain and feed efficiency is observed at 200 mg/kg	(125)	
THEO (0, 5, and 7.5 g/kg diet)	1–42 days	No effect on body weight, feed intake, or FCR	(131)	
20% volatile THEO (200 cc/1,000 L of water, 2×/day)	10–36 days	Lower mortalities, feed intake, and FCR in treatment group increase body weight	(160)	
THY (0, 200 mg/kg diet)	1–32 days	No impact on performance parameters	(22)	2014
EOs blend (THY 13.5 g, CIN 4.5 g per 100 g blend; 0, 50, 100 mg/kg)	1–42 days	No differences are observed in growth performance in *Salmonella spp*.-challenged broilers	(155)	
THEO (various concentrations)	1 to end of rearing period	No influence on growth performance	(58)	2016
EOs (25% THY and 25% CAR; 0, 60, 120, 240 mg/kg)	1–28 days	No growth performance changes are observed; FCR decreases between days 14 and 28.	(111)	
THY (0, 250 mg/kg)	During heat stress	Increases body weight gain, decreases FCR by 6% and 4%, respectively; increases carcass and breast percentages	(126)	
THP (0, 5 g/kg); THP-TUR (2.5 g/kg each)	1–42 days	Increases body weight in THEO|-only group; blend group had highest feed intake and lowest FCR	(152)	
THEO (0, 0.75% feed); THEO-TUR-coriander blend (0.25% each)	1–42 days	No significant growth performance differences	(161)	
Encapsulated phytogenic additive (100 mg/kg, including THY)	1–42 days	Increases body weight and weight gain by day 42; improves FCR during finisher phase	(162)	
THEO (0, 1, 1.5, and 2 g/kg) and mannanoligosaccharides	Rearing period in hot climate	Reduces feed intake in 1 g/kg group; best FCR with 1 g/kg THEO and mannanoligosaccharides; no differences in productive efficiency	(134)	2017
THP (2, 5, and 8 g/kg; *T. vulgaris* with 50.48% THY)	1–42 days	Best growth and economic results with 5 g/kg; 8 g/kg group shows lowest revenue and highest feed cost for 1 kg live weight gain	(135)	
EOs blend (25 mg/kg), combined or not with saponins (46 mg/kg). THY concentration in the diet: 1 mg/kg feed	1–21 days	Numerical improvement in growth performance traits of all groups fed the phytogenic additive compared to control broilers during the starter period	(163)	
THP (0, 10, 20, and 30 g/kg diet)	1–28 days	All the groups supplemented with the dried THP show a better FCR than control. Greater in the 30 g/kg diet group	(136)	2018
THEO (0, 0.05, and 0.1% w/w)	1–28 days	No significant impact on growth performance parameters, though a slight decrease in slaughter weight was noted	(59)	2019
THEO (0.00%, 0.01%, 0.05%, and 0.1%, w/w)	32–60 days	Performance parameters are unaffected by THEO	(70)	
THEO (47.59% THY; 0, 3 g/kg)	1–28 days	Higher daily gain and feed intake; lower FCR in broilers fed diets contaminated with mycotoxins	(137)	
EOs blend (THY 13.5%, CIN 4.5%; 0, 50, and 100 mg/kg)	11–42 days	No significant effect on final body weight, weight gain, growth rate, feed intake, or FCR	(142)	
THEO (0, 0.25, 0.5, 1%)	1–42 days	Increases weight gain and reduces FCR by 0.5% and 1% THEO	(164)	
THY (1 g/L/day, containing 15% THY) and amoxicillin (48 mg/L of product containing 700 mg/g amoxicillin) in water	2 weeks	Chickens in both THY and amoxicillin groups show significantly higher body weights than controls, with the THY group showing the lowest consumption index. THY positively enhances zootechnical performance	(84)	2020
THY (300 mg/kg diet), along with CAR and EUG as components of an EO blend	12 weeks	THY-supplemented groups in enriched cages show a 10% increase in egg production and improved egg weight and mass. Feed intake is lower with THY, and FCR improves across all periods. Eggshell strength, thickness, yolk color, and albumen height are all enhanced	(138)	
Encapsulated EOs with equal concentrations of THY and CAR (140 g/kg); dosages of 0, 60, and 120 mg/kg diet	28 days (post-coccidiosis challenge)	Significant increase in body weight gain and feed intake in broilers challenged with a higher dose of coccidiosis vaccine compared to challenged birds without EOs. EOs mitigated coccidiosis-induced reduction in weight gain and feed intake	(165)	
EOs blend (50, 100, 200, and 400 mg/kg; 42 days) with THY (3.05%) as the main compound and smaller amounts of CAR and CIN	42 days	A quadratic increase in body weight gain is observed during days 1 to 21 with EOs supplementation, suggesting optimal growth performance at moderate levels of the blend	(19)	2021
THEO, OEO, and other THY-containing EOs (star anise, rosemary at 25 mg/kg; THY concentration: 1.02 mg/kg diet) and saponins (46 mg/kg), alone and in combination	42 days	No significant performance differences during the starter period. During the grower and overall periods, all supplemented groups show higher weight gain than controls, with improvement of FCR in the EOs plus saponins group	(139)	
EOs from *Lippia origanoides* (0, 80, and 150 ppm) and zinc bacitracin (50 ppm)	Duration not specified	All treatments improve FCR more than the control group. 150 ppm EOs group: improve egg production, egg mass, and enhance external and internal egg qualities, including shell thickness and yolk color	(166)	
**Lyining hens**				
THP (0, 3, 6, or 9 g/kg)	36–52 weeks of age	No significant differences in body weight change, feed consumption, or FCR, but 3–6 g/kg THEO increase egg mass and weight	(128)	2015
THEO (45–50% THY) and peppermint (menthol); each at 100 mg/kg diet, individually and combined	56 days	Significant interactions between EOs on feed FCR, egg production, and egg mass. Combined EOs increase egg production and egg mass and reduce FCR compared to the basal diet	(140)	2016
THEO (THY concentration not reported); 0.9% of diet.	Not specified	THEO supplementation improves FCR, egg production, and egg output. Egg quality traits are significantly enhanced	(1)	2017
**Quails**				
THY: 400 mg/kg diet	12 weeks (from 4 to 16 weeks old)	THY supplementation does not significantly affect growth rate, final body weight, or egg production parameters, but increases hatchability	(167)	2018
THEO (35.40% THY) and savory EOs (33.06% THY); dosages of 200, 300, and 400 ppm	Not specified	A decrease in feed intake is observed in the 400 ppm THEO group, with a notable improvement in FCR at this dosage. Body weight gain remains unaffected by treatments	(141)	
THY; 0, 2, 4, and 6.25 g/kg diet	1 month	No significant differences in daily feed intake between treatments	(71)	2019
THEO (THY concentration not reported)	8 weeks	THEO treatment significantly improves average daily gain and FCR	(80)	
**Pigeons**				
THY: 40 mg/kg body weight in feed	15-day post-infection with *Eimeria labbeana*	Body weights of squabs treated with THY are significantly higher than controls on day 8 post-infection with *E. labbeana*. Treated groups show greater body weight gain than the untreated group	(143)	2020

**Table 17:** Productive performance in mammals fed with a diet supplemented with a THY source.

**Species**	**THY source and concentration**	**Duration**	**Main findings on productive parameters**	**References**	**Year**
	Encapsulated EO blend (50–150 g/T feed)	35 days	Improves weight gain and FCR above 100 g/T	(18)	2012
Pigs	THY (0.0067% and 0.0201%)	7 days	THY supplementation shows no significant effect	(168)	2014
	EO blend with 13.5% THY, 4.5% CIN (0.025%)	28 days	Increases average daily gain compared to control; similar performance to high-energy diet groups	(121)	
	EOs blend (30 mg/kg; 10% THY, 0.5% EUG, and 0.05% piperine)	56 days	EOs blend improves growth performance compared to control and THY-only groups	(127)	
	*Nigella* seed EO (THY concentration not reported, 0.5, 1, and 1.5 g/kg feed)	45 days	Greater growth is observed with 1 g/kg supplementation; no significant effect on feed intake or FCR	(21)	2015
	THY with BA (100 mg/kg or 200 mg/kg THY)	42 days	Tendency for lower feed-to-gain ratio with 2,000 mg/kg BA + 100 mg/kg THY	(133)	
	THY-CAR blend (0 and 100 mg/kg)	7 days	No significant differences in daily gain, feed intake, or FCR	(15)	2017
	THY (0.01% w/w)	Not specified	Decreases weight gain, no effect on feed efficiency, backfat thickness, or loin eye area	(122)	2020
	Microencapsulated THY (0, 25.5, 51, 153, and 510 mg/kg feed)	14 days	No significant differences in body weight, feed intake, daily gain, or FCR among treatments	(169)	
	*Lippia origanoides*-*E. caryophyllata* EOs (67–100 mg/kg)	56 days	Increases backfat thickness, selling weight, daily weight gain, and lower FCR	(20)	2021
	Microencapsulated THY (0.6 g/kg) or THY-fumaric acid blend (0.6 g/kg + 0.9 g/kg)	21 days	Improve FCR with microencapsulated THY and THY-fumaric acid blend	(170)	
	10% THY, 10% CIN, 10% d-limonene, 7.5% CAR (100–400 mg/kg)	42 days	No effect in early phases; higher gain-to-feed ratio in Starter II phase with 200 mg/kg and 400 mg/kg doses	(112)	2023
Dairy cattle	THEO (50 mg/kg) and THY (50 mg/kg)	28 days	No significant effects on milk yield or corrected milk (fat or energy)	(171)	2021
Rabbits	THY (3 and 6 mg/kg/d)	8 weeks	No significant differences in body weight gain or feed intake	(112)	2016
	THY (250 mg/kg feed)	21 days	No significant effect on body weight, weight gain, or FCR	(120)	2020
	THY (0, 100, 200, and 300 mg/kg)	6 weeks	No significant effects on FCR, feed intake, or mortality; meat from the 200 mg/kg group was more tender	(172)	2021
Blue foxes	THY (0, 100, 200, and 300 mg/kg)	30 days	The addition of 200–300 mg/kg THY to diets increases the final weight	(150)	2024

**Table 18:** Productive performance in fish fed with a diet supplemented with a THY source.

**THY source and concentration**	**Duration**	**Main findings on productive parameters**	**References**	**Year**
Phytogenic feed additive containing THY (0 and 1 g/kg)	8 weeks	Phytogenic supplementation improves feed efficiency. Body weight gain is not affected	(114)	2012
THEO (0, 500, 1,000, and 1,500 mg/kg)	60 days	Fish with THEO diets show significantly higher weight gain percentages and specific growth rates than control	(115)	2015
THEO (0.5%, 1%, 1.5%, and 2%)	56 days	THEO supplementation boosts growth rate, peaking at 1.5%.	(90)	2017
THY (1 and 2 ml/kg diet)	75 days	Dietary supplementation with 1 ml THY/kg diet significantly improves growth performance	(2)	2018
THEO (THY = 38.74%; 0, 0.5, 1, and 2 ml/kg feed)	2 months	0.5 ml/kg feed THEO yields significantly better weight gain and specific growth rate	(14)	2019
THP (2%; THY concentration not specified)	30 days	THP administration normalizes growth performance in fish exposed to lambda cyhalothrin insecticide	(13)	2020
THY (1, 1.5, 2, and 2.5 g/kg)	60 days	Fish fed THY-supplemented diets improve weight gain and FCR, with best results at 1 g/kg; protein content increases significantly in groups with 2–2.5 g/kg	(146)	2022
THEO (THY = 55.9%; 0 and 50 mg/L)	N/A	No differences in feed consumption	(147)	

#### 3.6.11 Effects of THY on digestibility, fermentation, and fatty acid profile

Studies consistently show that THY and THEO-based additives positively affect these parameters in certain animal species, though results vary based on dose and the target animal species. Table 19 synthesizes recent research findings on the impact of THY and THEO-based supplements on nutrient digestibility, microbial fermentation, and fatty acid composition. This summary includes studies on various livestock animals and pets, detailing each study's design, THY dosage, treatment duration, and key results.

**Table 19:** Effects of THY- and THEO-based supplements on digestibility, fermentation, and fatty acid profile in various animal species.

**Animal species**	**Treatment/THY dose**	**Duration**	**Main findings**	**References**	**Year**
Pigs	EOs blend (18% THY and CIN, 0.01%)	35 days	Increases dry matter and crude protein digestibility compared with control	(18)	2012
	THY (0.0067% or 0.0201%)	7 days	No significant effect on fermentation products in the gut	(168)	2014
	EOs blend (13.5% THY and 4.5% CIN, 0.025%)	28 days	Enhances apparent digestibility of dry matter, crude protein, and energy compared to control	(121)	
	THY (100–200 mg/kg) + BA (1,000–2,000 mg/kg)	42 days	Higher butyric acid concentrations and lower ammonia nitrogen in cecal content	(133)	2015
	*Lippia origanoides-E. caryophyllata* EOs (47.5% THY)	N/A	Increases cecal volatile fatty acids in treated groups	(20)	2021
Dogs	Yeast cell wall + OEO (0–3 kg/ton)	20 days	Reduces dry matter digestibility, lowers fecal ammonia	(17)	2023
Rabbits	THY (250 mg/kg)	21 days	Increases oleic, monounsaturated, and docosapentaenoic fatty acids in muscle	(120)	2020
	THY (0, 100, 200, and 300 g/T)	Days 35–77	Increases *Lactobacilli* and decreases *E. coli* in cecal microbiota	(172)	2021
Dairy cows	TEHO and THY (50 mg/kg)	28 days	No changes in volatile fatty acids or NH_3_	(171)	2021
Goats	THY (0–600 mg/L)	24 h	Reduces total gas and methane production and improves rumen fermentation characteristics	(173)	2020
Blue foxes	THY (0, 100, 200, and 300 mg/kg)	30 days	The addition of 100 mg/kg THY to the diet significantly increases organic matter digestibility and crude protein digestibility	(150)	2024
Broiler chickens	THY-CAR blend 1:1 (0, 60, 100, and 200 mg/kg)	N/A	Decreases saturated fatty acids and increases PUFAs in serum and thigh	(125)	2013
	THY (200 mg/kg)	0–32 days	Tendency to decrease propionic and butyric acid concentrations in cecal content, with no effect on molar ratios of short-chain fatty acids	(22)	2014
	Encapsulated phytogenic additive (100 mg/kg)	42 days	Increases digestibility of crude protein in ileum	(162)	2016
	EO blend with saponins (THY at 1 mg/kg)	N/A	Improves protein and amino acid digestibility in ileum; transcriptomic analyses indicate enhanced macromolecule uptake and metabolism	(163)	2017
	THEO (0.05–0.1%)	Days 0–28	No significant differences in fatty acid profile of breast muscle	(59)	2019
	Encapsulated THTY-CAR (0–120 mg/kg)	N/A	Decreased volatile fatty acids in coccidiosis-challenged chickens	(165)	2020
	EOs blend (50–400 mg/kg)	42 days	Higher digestibility of dry matter, gross energy, and ether extract at all levels; crude protein digestibility improves at high doses	(19)	2021
Broiler chickens feed	THY (400 mg/kg)	N/A	No changes in fatty acid composition	(167)	2017
Quail	THY (0.0016 mol/day)	Incubation period	Polyunsaturated fatty acids are provided to embryo at day 4 for synthesis/deposition in membranes	(174)	2017
	THY (0–6.25 g/kg)	28 days	Decreases saturated and increases PUFAs in high-dose groups	(71)	2019

#### 3.6.12 THY effects on gut microbiota

THY supplementation has been shown to positively impact gut health by modulating microbial populations, often reducing pathogenic bacteria, such as *E. coli* and coliforms, while supporting the growth of beneficial microorganisms like *Lactobacillus spp*. and other lactic acid bacteria. Changes in microbial diversity have also been observed, with certain studies reporting an increased abundance of beneficial taxa following THY supplementation. However, the effects of thymol can vary depending on factors such as dosage, species, and the specific gut region studied, with some reports showing no significant shifts in microbiota composition. Overall, evidence suggests that THY's influence on intestinal microbiota is context-dependent and may differ across animal species, including fish, pigs, poultry, and dogs. Table 20 summarizes findings from studies evaluating these effects.

**Table 20:** Summary of THY's effects on gut microbiota across animal models.

**Animal model**	**Supplement composition**	**Dosage/duration**	**Main findings on microbiota**	**References**	**Year**
Dogs	Yeast cell wall + OEO	1.5 and 3 kg/ton for 20 days	Increases *Blautia spp*. and *Fecal ibacterium spp*., decreases *Streptococcus spp*. in feces; greater bacterial diversity	(17)	2023
Weaned piglets	EOs blend (18% THY + CIN)	0.01% for 35 days	Improves fecal scores; reduces *E. coli* in cecum, colon, rectum; increases *Lactobacilli-E. coli* ratio	(18)	2012
	Encapsulated EOs (THY + CIN)	0, 50, 100, or 150 g/T of feed	Decreases *E. coli* counts in feces	(149)	
	THY	0.0067% or 0.0201% for 7 days	No significant effect on cecal anaerobes	(168)	
	THY + BA	THY: 100–200 mg/kg + BA: 1,000–2,000 mg/kg) for 14 days	Higher *Lactobacillus spp*. in ileum and *Bacillus spp*. in caecum	(133)	2015
	THY-CAR blend (1:1 ratio)	100 mg/kg for 14 days	Decreases *Enterococcus spp*. and *E. coli*; increases *Lactobacillus spp*.	(15)	2017
	*Lippia origanoides*-*E. caryophyllata* EOs (47.5% THY)	67 mg/kg; blends up to 100 mg/kg	No effect on *Enterobacteria* or *Lactobacillus spp*.	(20)	2021
	EOs blend (10% THY)	0–400 mg/kg for 42 days	No effect on microbiota in intestinal segments	(123)	2023
Goats	THY	600 mg/L	Alters bacterial community	(173)	2020
Blue foxes	THY	0, 100, 200, and 300 mg/kg for 30 days	THY increases the abundance of *Bifidobacterium spp., Fusobacterium spp*., and *Allobaculum spp*. in the gut	(150)	2024
Fattening rabbits	THY supplementation	Not specified	Higher *Lactobacillus spp*. and *Bifidobacterium spp*. populations	(16)	2021
Broiler chickens	THEO	Not specified	Reduction in pathogenic bacteria, promotion of beneficial microbes	(70)	2019
	THEO	0.5% and 1% for 42 days	Decreases microbial population in cecal content	(164)	2019
Microencapsulated THY + fumaric acid	0.6 g/kg for 21 days	Increases *Bacteroidetes, Bacillaceae*, and *Rikenellaceae*; decreases *Pseudomonadaceae* in cecal content	(170)	2021
Ducks	EOs blend (13.5% THY and 4.5% CIN)	11–42 days	Reduces cecal coliforms, total aerobes, and lactose-negative *Enterobacteria*	(142)	2019
Quail	THEO (35.4% THY)	Different levels	Lower coliforms in supplemented groups compared to antibiotic group; *Lactobacilli* unaffected	(80)	2019
Turkey	THY or EOs blend with THY	30 mg/kg for 56 days	Increase lactic acid bacteria, decreased coliforms in caecum; no effects in ileum	(127)	2014
Rainbow trout	Phytogenic additive (6 g/kg THY)	1 g/kg for 8 weeks	Lower anaerobe and *Lactobacillus* counts	(114)	2012

#### 3.6.13 THY effects on intestinal permeability, tight junctions and gastric mucosa gene expression and modulation of the endocannabinoid system in various animal models

In various animal models and *in vitro* systems, researchers assessed how THY influences tight junction proteins, nutrient transport genes, markers of epithelial integrity, and components of the endocannabinoid and chemosensing systems under both normal and inflammatory conditions. These studies collectively investigated THY's potential for enhancing gut function, maintaining epithelial resilience, and modulating signaling pathways involved in gastrointestinal homeostasis. Table 21 provides a comprehensive overview of key studies, detailing the effects of THY on target gene expression profiles related to barrier function (e.g., claudin-1, occludin), ion transport, gastrointestinal maturation (e.g., somatostatin, peptide transporter 1), and the endocannabinoid system, including cannabinoid receptors and enzymes involved in endocannabinoid biosynthesis and degradation.

**Table 21:** Effects of THY on intestinal permeability, tight junctions, gastric mucosa gene expression, and modulation of the endocannabinoid system in various animal models.

**Species**	**Target tissues**	**Treatment/dosage**	**Genes/proteins affected**	**Key findings**	**References**	**Year**
Pigs	Oxyntic and pyloric mucosa	THY, 50 mg/kg BW (oral)	Somatostatin, peptide transporter 1, calpain 9, Ion transport genes (TRP channels, K+ channels)	THY upregulates genes associated with gastric function and downregulates ion transport genes, suggesting potential support for gastric maturation	(175)	2014
	Jejunal mucosa (weaned piglets)	THY-CAR (100 mg/kg diet)	Occludin, ZO-1	No significant effect on occludin or ZO-1 expression in the jejunum, suggesting limited impact on these tight junction proteins in weaned piglets	(15)	2017
	IPEC-J2 cells (LPS challenge)	THY, 50 μM (pre-treatment)	ZO-1, Claudin-3	No significant changes in ZO-1 and claudin-3 with THY under inflammation. Pre-treatment with THY restores TEER and reduces FITC-dextran leakage, indicating preservation of epithelial integrity	(25)	2019
	Duodenal and Ileal mucosa	Microencapsulated THY (0, 25.5, 51, 153, and 510 mg/kg feed)	Endocannabinoid system and gut chemosensing markers. CB1, CB2, TRPV1, OR1G1, DGL-α/β, FAAH	THY modulates CB1, CB2, and related markers, influencing gut chemosensing and cannabinoid signaling pathways	(169)	2020
Broiler chickens	Ileum (challenged with *C. perfringens*)	THY-CAR (0, 60, 120, and 240 mg/kg diet)	Down: claudin-1, occludin; unaffected: mucin-2	Higher EOs doses reduce lesion severity, with THY potentially protecting against barrier disruption caused by *C. perfringens*	(111)	2016
	Jejunum	THP (0, 2, 5, and 8 g/kg)	Up: mucin-2	THY upregulates mucin-2 mRNA in jejunum	(135)	2017
	Jejunum and ileum	THY (50, 100, 200, and 400 mg/kg diet)	Up: nutrient transporters (GLUT2, SGLT1, SLC38A, SLC79A, SLC27A4), TJP1	THY supplementation upregulates nutrient transport and barrier function genes, enhancing gut health indicators in broilers	(19)	2021
	Chicken enteroids; 18-day-old embryos (LPS-induced inflammation)	THY: 10 ppm and THEO: 20 ppm (6-h exposure)	Up: occludin, ZO-1	Restore tight junction gene expression	(113)	2023

#### 3.6.14 THY effects on intestinal and hepatic morphological parameters

Data revealed consistent benefits of THY supplementation on gut morphology, as indicated by increased villus height and villus-to-crypt ratios in most animal models. These morphological improvements suggest enhanced absorptive capacity, which could contribute to better nutrient uptake and overall gut health. Concerning the liver, published data indicate that higher THY doses or its prolonged exposure may cause mild, reversible changes. Table 22 provides an overview of studies investigating the effects of THY on intestinal and hepatic morphological parameters across various animal models, including weaned piglets, broiler chickens, quail, pigeons, and rabbits.

**Table 22:** Summary of studies evaluating the effects of THY on intestinal morphology in various animal models and hepatic morphology in quails.

**Species**	**THY dosage/composition**	**Study duration**	**Morphological parameters**	**Significant findings**	**References**	**Year**
**Intestinal morphology**						
Weaned piglets	0.01% EO blend (18% THY and CIN)	35 days	Villus height depth ratio in jejunum	Increases ratio in jejunum for EO blend group	(18)	2012
	0.025% EO blend (13.5% THY and 4.5% CIN)	28 days	Villus height	Increases villus height with EO blend	(121)	2014
	THY (100–200 mg/kg) + BA (1,000–2,000 mg/kg)	14 days	Villus height depth ratio in jejunum, ileum	Highest ratio at 2,000 mg/kg BA + 100 mg/kg THY	(133)	2015
	THY-CAR (100 mg/kg)	7 days	Intestinal morphology	No differences between groups	(15)	2017
	*Lippia origanoides*-*E. caryophyllata* EOs (47.5% THY)	Not specified	Jejunal goblet cells, villus height depth ratio	Increases goblet cells, improves mucus adhesion, and enhances villus/crypt ratio	(20)	2021
Blue foxes	THY (0, 100, 200, and 300 mg/kg)	30 days	Villus height, crypt depth	Increases villus height (duodenum, ileum), crypt depth (jejunum), and villus/crypt ratio (ileum)	(150)	2024
Fattening rabbits	THY supplementation	Not specified	Villus height, crypt depth	Increases villus height, reduces crypt depth	(16)	2021
Broiler chickens	Dried THEO (0.75% feed)	Not specified	Villus height, crypt depth	Increases villus height and crypt depth	(161)	2016
	60–120 mg/kg encapsulated EO (50% THY)	20 days	Villus height depth ratio in ileum	Highest ratio at 120 mg/kg EO	(165)	2020
	25 mg/kg EO (THY = 1.02 mg/kg) + saponins	42 days	Villus height, crypt depth	Increases villus height and villus/crypt ratio	(139)	2021
	0.6 g/kg THY + fumaric acid blend	35 days	Ileal villus height depth ratio	Increases ratio after fasting stress simulation	(170)	
Quail	200–400 ppm THEO and savory EO (35–33% THY)	Not specified	Villi height in duodenum, jejunum, ileum	Increases villi height, reduces crypt depth in supplemented groups	(141)	2018
	2–6.25 g THY/kg feed	30 days	Liver morphology	High occurrence of steatosis, sinusoidal dilation, vascular congestion, low necrosis rate	(71)	2019
	80–150 ppm *Lippia origanoides* EO	Not specified	Villus height, crypt depth	Increases villus height, reduces crypt depth, improves ratio of 150 ppm	(166)	2021
Pigeons	40 mg/kg THY in feed	15 days	Intestinal morphology, inflammatory response	Intact villi reduce coccidian stages, mononuclear cell infiltration	(143)	2020
**Hepatic morphology**						
Quail	2–6.25 g THY/kg feed	30 days	Liver morphology	High occurrence of steatosis, sinusoidal dilation, vascular congestion, low necrosis rate	(71)	2019

#### 3.6.15 Other effects of THY and THEO

Recent studies have explored various additional effects of THY across diverse biological systems, though research remains limited in most cases. These effects are summarized below:

##### 3.6.15.1 Anticarcinogenic effects

Thymol (THY) has demonstrated anticancer activity in human cell lines and, in preclinical models, has shown protective effects against colon cancer. A 2021 study (7) in rats exposed to 1,2-dimethylhydrazine and a high-fat diet found that THY reduced serum levels of tumor markers (CEA, CA 19-9) and caspase-3, attenuated oxidative stress and inflammation in colonic tissue, and improved histopathological alterations, supporting its potential as a chemopreventive agent. Although these findings come from experimental models, they may have particular relevance for companion animals, such as dogs and cats, where colorectal cancer represents a clinical concern and research remains limited.

##### 3.6.15.2 Behavioral effects

Several studies have investigated the potential anxiolytic properties of thymol (THY) in experimental animal models. THY appears to modulate stress- and fear-related behaviors, positioning it as a promising natural compound for managing anxiety. Although limited in number, the available studies, conducted primarily in avian (176) and rodent models (23), have assessed behavioral responses to experimentally induced stress, reporting reductions in anxiety-like behaviors such as excessive struggle and altered exploratory activity. Notably, these effects occurred without compromising general locomotor function, suggesting a selective action on anxiety-related pathways.

##### 3.6.15.3 Anesthetic properties

THY and THEO exhibit dose-dependent anesthetic properties in various fish species, such as silver catfish, common carp, and tambaqui, making them promising alternatives to conventional anesthetics in aquaculture, as their efficacy depends on both concentration and species-specific sensitivity. It has been shown that THY acts *via* GABAA_AA receptors, although through mechanisms independent of benzodiazepine binding sites (75). The anesthetic potential of THY in common carp has been confirmed, although with slower induction and prolonged recovery times (117). Similarly, THEO has been reported to induce anesthesia in tambaqui, with higher doses associated with faster induction but extended recovery periods (147). These findings support the utility of THY as a natural anesthetic agent, although interspecies variability and concentration-dependent effects must be taken into account.

##### 3.6.15.4 Wound healing

In rodent models, THY has demonstrated significant wound healing properties across different formulations and wound types. A study using collagen-based dressing films infused with THY reported enhanced wound retraction at days 7 and 14, along with improved granulation tissue and increased collagen density, suggesting accelerated and higher-quality tissue regeneration (105). Similarly, a THY-enriched bacterial cellulose hydrogel (1%) applied to third-degree burn wounds resulted in faster re-epithelialization, reduced inflammation, and improved collagen deposition over a 25-day evaluation period (46). These findings highlight the therapeutic potential of THY in promoting wound healing in preclinical models.

##### 3.6.15.5 Bone loss

THY has been shown to exert significant anti-osteoclastogenic and bone-protective effects. *In vitro*, THY inhibits osteoclast differentiation in a dose-dependent manner, as evidenced by a reduction in both the number and size of TRAP-positive multinucleated cells. *In vivo*, THY administration in mice effectively prevents lipopolysaccharide (LPS)-induced bone loss, improving bone microarchitecture and reducing osteoclast counts (26). These findings underscore THY's potential in protecting bone integrity and modulating osteoclastogenesis.

##### 3.6.15.6 Ovarian function

THY was evaluated for its effects on follicular activation, stromal cell protection, and collagen fibers in bovine ovarian cortical tissues. THY increased the percentage of normal follicles and improved follicular activation, collagen fiber density, and stromal cell density (129). These findings suggest that THY promotes follicular activation and helps maintain ovarian tissue integrity, indicating a potential protective effect on ovarian function.

##### 3.6.15.7 Halitosis management

A clinical trial in dogs diagnosed with oral malodor shows that a THY- and menthol-containing gel effectively reduces bad breath by targeting malodor-producing bacteria (8).

## 4 Discussion

This systematic review highlights THY's potential as a bioactive compound in veterinary medicine due to its diverse pharmacological properties and comparatively low toxicity across various animal models. Key areas of THY's application include its antimicrobial, anti-inflammatory, antioxidant, and therapeutic roles in managing parasitic infections, gut health, and productive performance in livestock. The results presented in this review support the potential of THY as a bioactive compound with diverse health and productivity benefits across animal models and cellular studies. Researchers have demonstrated THY's efficacy in enhancing health outcomes, improving productivity, and mitigating the adverse effects of environmental and metabolic stress in different species. However, the effectiveness of THY appears to be contingent upon factors such as dosage, form of administration, and the specific physiological context of the target species.

THY's cytotoxicity varies widely across cell types, with LC_50_ or IC_50_ values ranging from 0.002 mg/mL (13.32 μM) in canine bone marrow stromal cells and enterocyte-like cells (30) to 362 μM in rat splenocytes (40). This range underscores the need for cell-specific considerations in therapeutic applications. Canine and porcine enterocytes are moderately sensitive to THY, with toxic concentrations above 100 μM in pig IPEC-J2 cells (25) and 0.05 mg/mL (333 μM) in canine hepatocytes (33), while EOs from various species show no toxicity in porcine hepatocytes (32), suggesting possible mitigating effects from other constituents. THY is relatively safe in immune cells and fibroblasts at low concentrations, with no significant cytotoxicity observed in murine macrophages up to 40 μg/mL (266.5 μg/mL); however, higher doses (665.94 μM) reduce cell survival (3). Fibroblasts (NIH-3T3) show only a slightly reduced viability at increasing THY doses (46), suggesting the obtainment and use of effective doses may be feasible. Tolerance to THY also varies in renal and mammary cells, with moderate toxicity (IC_50_ = 300 μM) in Vero cells (35) and no significant toxicity in mammary epithelial cells from mice and cattle, even at high concentrations (24, 28), thereby highlighting THY's potential for low-dose veterinary applications prioritizing renal and mammary health. *In vivo* toxicity data show species-specific, dose-dependent effects, corroborating the *in vitro* cytotoxicity findings. For instance, THY's LD_50_ in rodents is 1350.9 mg/kg, while its derivative THY acetate has a much higher LD_50_ (4,144.4 mg/kg) (47), suggesting how structural modifications can reduce acute toxicity. Extended THY exposure at lower doses (10–40 mg/kg over 30 days) induces immunotoxic effects, affecting ATP hydrolysis pathways and triggering inflammation (48), linking back to *in vitro* data showing stress responses in immune cells. Further *in vivo* studies on embryotoxicity and estrogenicity indicate that THY (0.5 mg/kg) in chickens induces developmental abnormalities such as curled claws and everted viscera, alongside weak estrogenic activity, although without evident mutagenic effects (50). These findings suggest that THY could interfere with organogenesis and hormonal regulation at higher concentrations. The alignment between *in vitro* and *in vivo* results demonstrates that while *in vitro* assays highlight potential risks, *in vivo* data provide a more comprehensive view of THY's systemic effects, including inflammation and developmental impacts, emphasizing the need for cautious application in sensitive contexts.

THY pharmacokinetics show species-specific variations in absorption, half-life and bioavailability, significantly influenced by administration route, encapsulation, and dosage. Understanding these profiles is essential for effective therapeutic use and minimizing adverse effects. Rodent studies indicate a rapid systemic absorption with short half-lives (~2.5–3 h) *via* intravenous and inhalation routes (49, 55). In rabbits fed with THY-containing diets, THY plasma levels correlate with concentrations found in the intestinal wall, suggesting a focus on gastrointestinal distribution (56). In broiler chickens, THY absorption increases in a dose-dependent manner with THEO supplementation, highlighting the avian gut's efficiency in absorbing lipid-soluble compounds such as essential oils and their components (57, 59). For pigs, encapsulated THY formulations extend half-life and bioavailability, enhancing intestinal targeting while reducing systemic exposure (60, 61). In dairy cattle, intramammary THY shows limited systemic absorption, supporting its use in mastitis with low milk residue risks (63, 64). These findings underline the need for tailored dosing and encapsulation to optimize THY's efficacy and safety across species.

Residue analysis also reveals species-specific THY dynamics. In dairy cattle, liver and milk retain THY residues post-treatment, suggesting a 72-h withdrawal period (63, 64). Broiler chickens show THY localization in the gut with minimal muscle residue, supporting its safe use as a feed additive (57, 70). In quails, THY dose-dependently accumulates in eggs, thus necessitating a withdrawal period to prevent residual concentrations (71). Pigs demonstrate limited systemic deposition, primarily in the gut, enhancing safety as a dietary supplement (61). Collectively, these findings suggest that THY, used within regulated doses, minimizes residue risks across animal products, making it a safe additive with appropriate withdrawal guidelines.

THY's interaction with xenobiotics, particularly veterinary drugs, remains underexplored but may significantly influence drug efficacy and metabolism in production animals. Known for its therapeutic benefits, THY may impact oxidative, reductive, and conjugative drug metabolism, potentially altering co-administered drugs' pharmacokinetics and pharmacodynamics. For example, THY altered ABZ's metabolism in lambs, reducing the C_max_ and AUC of ABZ sulfone. Notably, THY did not enhance ABZ's efficacy, but instead inhibited sulphonation and sulphoreduction processes, suggesting enzyme competition that may affect drug clearance (29). This highlights the need for research into THY's effects within multi-drug regimens, given its potential to influence therapeutic outcomes in production animals.

THY exhibits broad-spectrum antimicrobial activity, being effective against both Gram-positive and Gram-negative bacteria and making it valuable in addressing antibiotic resistance in veterinary contexts. THY exerts its antibacterial effects through multiple mechanisms, primarily by disrupting cell membranes. It integrates into the lipid bilayer of bacterial membranes, increasing permeability and leading to the leakage of essential intracellular components, ultimately causing cell death (72). Furthermore, THY inhibits bacterial quorum sensing, thereby reducing biofilm formation and virulence. Additionally, it damages bacterial DNA and RNA, impairing replication and transcription, and inhibits key metabolic enzymes such as ATPases and glycolytic enzymes, vital for bacterial energy production. These combined actions contribute to THY's effectiveness as an antibacterial agent. THY's antibacterial properties are thus not only due to membrane disruption but also to its interference with essential cellular processes, making it a potent antimicrobial compound. Effective concentrations depend on pathogen type, THY form, and application. Studies show that THY inhibits a range of pathogens, including those resistant to common antibiotics, at MICs of 0.01–0.32 mg/mL (77). THY demonstrates stronger antimicrobial effects when combined with CAR or CIN essential oils (5, 86), and its derivatives may be more effective than alternatives like EUG against *S. aureus* (51). The variability in MIC values reported across studies, such as 0.4 mg/mL for *Thymus numidicus* essential oils against *Pseudomonas* spp. (79) and 2.5–160 μg/mL for *Thymus* spp. against various pathogens (32), reflects differences in *Thymus* species and microbial species, with formulations and study conditions also potentially influencing the results. Concerns about resistance are limited with THY; studies found no resistance in *E. coli* or mesophilic gut flora post-treatment (84), though tolerance was observed in some cases (81). THY's efficacy against pathogens causing bovine mastitis (*S. aureus, E. coli, St. uberis*, and the algae *Prototheca bovis*) suggests it could serve as an alternative or adjunct to antibiotics (66, 87). While THY's antimicrobial utility is clear, the variability in MIC/MBC values underscores the need for standardized protocols in veterinary chemotherapy.

THY and THEO demonstrate varied antiviral effects, showing efficacy against certain viruses while presenting limitations with other ones. Studies reveal strong inhibitory effects on enveloped viruses such as HSV-1, where a 90% virion inactivation was noticed at an IC_50_ value of 7 μM (35). THY's antiviral action likely stems from its ability to destabilize viral envelopes or capsids, inhibiting viral replication and interfering with viral entry into host cells. In contrast, THY showed mixed results against non-enveloped viruses. For instance, it dose-dependently inactivates norovirus surrogates, while it shows minimal effects on HAV, thereby suggesting that non-enveloped viruses may resist THY's mechanism or require higher doses to be effective (38). The structural disruption of viral envelopes by THY supports its selective efficacy against enveloped viruses, while higher concentrations may be necessary for non-enveloped viruses. THY-enriched EOs demonstrated potent virucidal effects against feline coronavirus (FCoV-II), reducing titers by up to 3.25 log_10_ (88), indicating potential veterinary applications. However, there is a lack of effect on FCV and its effectiveness against lipid-enveloped viruses. THY's concentration-dependent antiviral effect and structural interaction with viral envelopes further emphasize its selective efficacy.

THY and THEO also exhibit broad-spectrum antifungal efficacy, impacting pathogenic, dermatophytic, and environmental fungi. Studies demonstrate consistent antifungal effects, with a significant inhibition of *Rhizopus oryzae* growth at 128 μg/mL (89); in addition, a strong growth inhibition was observed with various *Thymus* species (32). THY's antifungal action is primarily due to the disruption of fungal cell membranes. It alters fatty acid metabolism, reduces ergosterol content, and induces oxidative stress through the generation of ROS, leading to fungal cell death. THY's effectiveness in combination therapies is noteworthy; for instance, a THY-itraconazole-clarithromycin blend achieved 96% inhibition against *Pythium insidiosum*, suggesting synergy that could enhance conventional treatments and reduce dosage (9). This synergy could be beneficial for treating complex fungal infections, particularly in veterinary settings. Additionally, THY vapor has been shown to inhibit fungal growth and toxin production; as an example, it suppressed *A. flavus* growth and reduced aflatoxin B1 production through gene downregulation (10), thereby suggesting potential for THY in agricultural food safety to control both fungal growth and toxin contamination in stored grains.

THY's antifungal potential extends to veterinary contexts, particularly against dermatophytes and fungi linked to infections. THEO is effective at low concentrations (0.5–2.5 μg/μL) against skin-infecting fungi (91), while THY-rich THEO inhibited *Malassezia pachydermati*s, common in canine ear infections (92). This supports THY's suitability in topical treatments for dermatophytic infections in animals. Additionally, species-specific sensitivity to THY was observed; for instance, dietary THEO (0.5–2%) protected carp from *Saprolegnia spp*. (90), suggesting that dietary supplementation could protect against fungal infections in aquaculture. The versatility of THY application methods, including liquid and vapor forms, offers flexibility for use in diverse veterinary and agricultural practices, as noted for *A. flavus* (10).

THY and THY-containing EOs demonstrate strong antiparasitic properties against various ectoparasites, such as flies, ticks, mites, and mosquitoes, indicating high potential for pest control in veterinary and agricultural settings. Significant acaricidal effects have been noticed on ticks like *Rhipicephalus microplus* and *Ixodes ricinus*, with *Lippia gracilis* EOs displaying high efficacy (LC_50_ between 0.84 and 1.02) even on resistant strains (11, 94). THY's larvicidal and repellent actions were similarly effective, achieving over 90% repellency and 100% larvicidal activity against ticks (94), which supports its utility as a natural acaricide. Additionally, prolonged residual effects were observed in certain applications; for example, a THY-CAR blend provided 14-day protection against red mites (*Dermanyssus gallinae*) in poultry (95). Such sustained effects make THY viable for long-term pest control, reducing treatment frequency. THY has also shown ovicidal and larvicidal activity across ectoparasite life stages. For example, including THY in quail diets reduces housefly (*Musca domestica*) oviposition (4), which is beneficial for livestock environments where flies proliferate. THY's effects were enhanced when combined with other natural compounds like CAR and EUG, suggesting synergistic potential. Significant reductions in mosquito and tick populations were observed when using THY-based blends (6, 12), highlighting lower dosage requirements and improved animal tolerance compared to standalone treatments. Moreover, THY demonstrated comparable or superior efficacy to synthetic acaricides like permethrin against ticks (94), showing promise as an organic alternative for pest management in contexts where chemical residues are a concern. Beyond ectoparasites, THY exhibits broad-spectrum efficacy against protozoan and helminth parasites, with a potential for use as a natural anthelmintic in veterinary practices. THY has shown strong oocysticidal effects against protozoa such as *Eimeria spp*. and *Cryptosporidium spp*., which are key pathogens in poultry. Specifically, THY disrupts oocyst wall integrity, causing parasite death with LC_50_ values of 1.66 mg/mL for *Eimeria* (96) and <0.4 mg/mL for *Cryptosporidium* (104). These findings support THY's potential as a natural disinfectant in poultry farming, reducing reliance on chemical agents that may leave residues in food products. Significant anthelmintic activity was also observed against parasitic nematodes, especially *Haemonchus contortus*, a prevalent gastrointestinal parasite in ruminants. THY's efficacy extends to cestodes like *Echinococcus granulosus*, with a reduction of cyst infectivity following a THY-induced structural damage in protoscoleces (98), suggesting THY's utility in treating cestode infections, especially where drug resistance is a concern. THY has also been proven effective against *Leishmania spp*., which may have implications for treating leishmaniasis in animals. In particular, THY derivatives showed a significant activity against *Leishmania infantum chagasi* promastigotes (3), while THY alone reduced parasite loads in infected hamsters (103). These findings suggest THY's therapeutic potential for zoonotic diseases, possibly offering a safer, cost-effective alternative to traditional antileishmanial drugs. Finally, THY interacts with synthetic anthelmintics such as ABZ; indeed, THY inhibits hepatic ABZ metabolism, thereby affecting its activation (65). While this interaction could support co-treatment strategies, it underscores the need for research into THY's pharmacokinetic interactions to prevent unintended efficacy reduction or toxicity, particularly in livestock where drug combinations are prevalent.

In terms of mechanisms of action, THY's anti-inflammatory and antioxidant effects are multifaceted and involve various molecular pathways and cellular processes. As to anti-inflammatory effects, THY exerts its influence through several mechanisms. The inhibition of MPO activity (105) is one key mechanism, preventing leukocyte infiltration and reducing the oxidative damage associated with inflammation. THY also reduced inflammation in pleurisy models (43), although its effect on cell migration varied and hints at concentration-specific responses. Additionally, THY modulates cytokine production, notably increasing the ratio of IL-10 to IL-2, which supports a Th2-dominant anti-inflammatory response (40). This modulation of immune responses is likely mediated by THY's ability to interfere with NF-κB and MAPK signaling pathways, which are crucial in the regulation of pro-inflammatory cytokines, such as TNF-α, IL-6, and IL-1β (24). Systemically, THY effectively reduced inflammation markers in liver and vascular tissue models. For example, it decreased TNF-α in a liver inflammation model (106), while reductions in VCAM-1, MCP, and C-reactive protein were noticed in a high-fat diet-induced inflammation model in rabbits (112), highlighting THY's broad efficacy in both tissue and systemic inflammation. Studies in broilers and piglets (15, 111) revealed a reduction of pro-inflammatory markers, showing potential applications of THY in livestock where bacterial infections and stress-induced inflammation are relatively common. Additionally, THY reduced lung inflammation markers and enhanced antioxidant defenses through Nrf2 and HO-1 pathways (108), while a dose-dependent reduction of the inflammatory response was observed in a model of gastric ulcer (109), underscoring THY's therapeutic versatility. Overall, these findings suggest that THY can effectively manage both acute and chronic inflammatory conditions, with evidence supporting dose-dependent optimization for enhanced therapeutic outcomes.

In terms of oxidative stress, THY's antioxidant activity is attributed to its ability to scavenge free radicals and enhance endogenous antioxidant defense systems. THY reduces MDA, a marker of lipid peroxidation, while simultaneously boosting the activity of antioxidant enzymes such as SOD and GPx (114, 117). This dual effect helps to reduce oxidative damage to cellular structures, particularly in tissues exposed to environmental stressors or inflammatory responses. In aquatic species, such as rainbow trout and Nile tilapia, THY supplementation demonstrated significant protection against lipid oxidation (2), suggesting its potential use in aquaculture to enhance fish health and stability. Similarly, THY supplementation in pigs and rodents has been linked to improved oxidative balance, with THY mitigating the oxidative damage caused by infections and pharmacological treatments, thus supporting its potential use in livestock management (106, 118). THY's antioxidant properties encompass its ability to reduce oxidative stress markers in high-fat diet-induced models, indicating its broad applicability in managing diet-induced oxidative conditions (112). Furthermore, THY's influence on oxidative stress pathways is not only limited to lipid peroxidation; it also affects reactive oxygen species (ROS) production. *In vitro*, THY's ability to reduce ROS in LPS-stimulated macrophages and intestinal cells confirms its direct role in modulating oxidative bursts, which are common in inflammatory responses. This further strengthens its potential as a therapeutic agent in conditions where both inflammation and oxidative stress are prevalent, such as colitis and gastrointestinal disorders (42). In poultry, THY increased antioxidant defenses (125), with dose-responsive trends improving resilience in broiler chickens and laying hens (1). THY's efficacy depends on botanical source, with some *Thymus* species offering higher radical scavenging activity than other ones (32). Overall, THY's ability to reduce MDA and increase antioxidant enzyme activity supports its role as a natural antioxidant in veterinary applications, making it a promising option for managing oxidative stress-related conditions across species.

Studies examining THY's impact on blood biochemical markers across animal models demonstrate beneficial yet variable responses, which are influenced by species, dosage, and formulation. Generally, THY has shown potential in enhancing blood lipid profiles and modulating immune functions, but optimized dosing is crucial given the variability observed among species. In terms of lipid profiles, THY supplementation reduced serum cholesterol, triglycerides, and LDL levels in rabbits and quail, while increasing HDL levels (112, 141). Similar lipid-lowering effects were observed in broilers, suggesting THY's potential role in managing lipid profiles and possibly supporting cardiovascular health in high-stress environments common to intensive farming (126). As far as blood biomarkers are concerned, in broilers and fish, THY showed immunostimulatory effects by enhancing hematological markers, particularly leukocyte and lymphocyte counts (90, 144). In broilers given THY, increased eosinophil, lymphocyte, and monocyte percentages have been noticed (138), which could enhance resilience to infections and reduce the need for antibiotics. In rainbow trout fed with a THY-supplemented diet, an increase in hematocrit, hemoglobin, and WBC counts was observed (146), suggesting an enhanced oxygen transport and immune function in fish. However, certain studies reported minimal changes in hemato-biochemical parameters, indicating that THY's physiological effects may vary across species and contexts. In ducks given THY supplementation, no significant impact on serum proteins or cholesterol levels was recorded (142), while minimal biochemical changes occurred with a 20% THEO (130), suggesting that specific formulations or lower concentrations may not elicit strong physiological responses. As mentioned above, species differences in the response to THY and THY-based EOs have been recorded, too. For instance, THY elicited dose-dependent effects on goat liver enzymes, where higher doses induced hepatic strain (14), underscoring the importance of cautious dosing to avoid liver stress with long-term use.

In poultry, THY and THY-based EOs showed indirect benefits on animal digestion; indeed, increasing trypsin, lipase, and protease activities were noticed in broilers (125, 139), indicating THY's potential to improve nutrient absorption, feed efficiency and the overall digestive process. Interestingly, THEO is beneficial in regulating glucose levels post-anesthesia in fish, which could help mitigate stress during handling and transport (147). However, contradictory results on lipid metabolism markers have also been observed, suggesting that age or health condition could influence digestive benefits (133).

THY's immune-modulating effects also demonstrate its broad applicability across different species. It is evident that THY can enhance immune function by increasing antibody titers, modulating cytokine responses, and improving immune cell function. A number of studies made in piglets (18, 121, 149) are an example of THY's ability to boost immune resilience through improvements in lymphocyte proliferation and elevated levels of immunoglobulins (IgA, IgM) and complement proteins. This is particularly valuable during growth phases or periods of stress, reducing vulnerability to infections. Similarly, poultry studies [e.g., (125) and (151)] showed that THY improves responses to vaccinations (e.g., against Newcastle disease) and reduces stress markers in broilers, enhancing overall health and vaccine efficacy. Additional research showed increased antibody titers for viral pathogens (21, 152), suggesting that THY could support vaccine efficacy and overall health. In aquaculture, THY's effects on immune health have also been demonstrated through an enhancement of lysozyme and catalase activities, which are critical components of innate immunity in species like rainbow trout (114) and Nile tilapia (2). THY also reduces bacterial load, including pathogens such as *Campylobacter spp*. and *Salmonella spp*., which are relatively common in poultry farming (155, 156). This reduction in bacterial infections is a key consideration in veterinary applications, especially in reducing antibiotic use in intensive farming systems. THY's potential to reduce mastitis in dairy cows, by reducing *S. aureus* adhesion (45), further exemplifies its antimicrobial benefits, especially in high-stress and high-density farming environments. In terms of dose-dependent effects, it is crucial to note that THY's efficacy in enhancing immune responses and reducing stress-related immune suppression is contingent on the optimal dosing. Studies with hens (128) and trout (14) show that lower or higher doses can either reduce efficacy or even suppress immune function, indicating the need for careful dose optimization to maximize its benefits. Furthermore, THY is promising in mitigating immunosuppression caused by environmental stressors. THY supplementation improved immune responses in broilers exposed to mycotoxins (137) and protected African catfish from pesticide-induced immunotoxicity (148). Additionally, THY's anti-inflammatory properties provide protective effects in models of induced tissue damage, such as reduced colonic inflammation in colitis models (42) and pancreatic protection in conjunction with non-steroidal anti-inflammatory drugs (153). In summary, THY's diverse immune-modulating properties, spanning from enhancing resistance to infections to alleviating stress-related immune suppression, underline its potential as a natural additive for improving animal health. This broad-spectrum efficacy, particularly in high-stress production environments, suggests that THY could reduce dependency on antibiotics, making it a promising agent for sustainable animal health management.

Data indicate that THY and THEO supplementation yield mixed results on productivity in various animal species, with effects largely dependent on dosage, species, and environmental conditions. For instance, 200 mg/kg of THY and CAR improved poultry feed efficiency and weight gain (125), possibly due to enhanced digestive enzyme activity and gut health. In broilers under heat stress, THY improved weight gain and feed FCR, underscoring its capacity to mitigate stress-related performance losses (126). However, other studies [e.g., (58) and (155)] found no significant impact on performance, thus suggesting that THY may be more effective in challenging environments than in optimal conditions. In egg-laying hens, THY combined with other EOs improved FCR, enhanced egg production and quality, and shell strength, maybe due to THY's antioxidant properties (1, 138, 140). For meat and dairy production, the findings were inconsistent. While some authors reported no effect on milk yield or composition in dairy cattle (171), other ones (18) observed weight gain improvements in pigs when using encapsulated THY (18), which may enhance nutrient absorption by targeting specific gut regions. In aquaculture, THY supplementation under stressful or pathogen-challenged conditions resulted in improved survival rates and growth. Its antimicrobial and immune-boosting properties appear beneficial under high pathogen loads, though effects on FCR and growth in unstressed conditions remain inconsistent. THY also enhances nutrient digestibility and gut microbiota composition across species. In monogastric animals, studies on piglets (18, 121), blue foxes (150), and broiler chickens (125, 163) showed increased digestibility of dry matter, protein, and energy, likely resulting from improved gut health. THY also affects fatty acid profiles: it provoked a reduction in saturated fatty acids and an increase in polyunsaturated fatty acids (PUFAs) in broiler meat (125), with similar trends observed in eggs, too (174). In ruminants, THY's potential to alter fermentation profiles is notable for sustainability. It is capable to Yu et al. (173) reduce methane production in an *in vitro* rumen model (173) and protozoa density in dairy cows without changes in pH or volatile fatty acids (171). Variable responses in volatile fatty acid profiles across species suggest a need for targeted research to optimize dosing and application methods for maximum productivity and environmental benefits. Overall, THY's effects on productivity, nutrient digestibility, and gut microbiota underline its potential as a natural additive in animal production systems. However, species-specific responses and mixed findings on fatty acid and methane profiles highlight the need for further studies to refine its application across livestock sectors.

Studies on THY supplementation reveal generally positive effects on gut microbiota across animal species, frequently reducing pathogenic bacteria like *E. coli* and coliforms while enhancing beneficial populations, such as *Lactobacillus spp*. This suggests THY may play a key role in gut health and microbiota balance, though outcomes depend on dosage, delivery method, and existing microbial communities. For example, in canines, THY with yeast cell wall increased beneficial bacteria (17), including *Blautia* and *Faecalibacterium*, while reducing *Streptococcus*, underscoring THY's potential to boost gut health and immune resilience. In piglets, Li et al. (18) and Diao et al. (133) reported reduced *E. coli* and increased *Lactobacillus*, supporting gut barrier function and nutrient absorption, which are especially crucial post-weaning. In poultry, THY consistently improved microbiota, reducing harmful bacteria while boosting beneficial ones, which can enhance growth and feed efficiency. In pigs, Abdelli et al. (170) observed that microencapsulated THY increased beneficial bacterial families (e.g., *Bacteroidetes* and *Bacillaceae*), while reducing pathogenic *Pseudomonadaceae*. These findings indicate THY's potential to foster a balanced gut microbiome. Studies in blue foxes, rabbits and quail also show increases in beneficial bacteria, suggesting THY's utility as a natural alternative to antibiotics. However, its effects on aquatic species, such as trout, are inconsistent and require further research to understand species-specific responses.

THY also appears to support gut barrier integrity, especially in inflammatory contexts. For instance, it protects IPEC-J2 cells from lipopolysaccharide-induced permeability disruptions (25), suggesting it stabilizes tight junctions and limits inflammatory damage to epithelial cells. Although changes in proteins like ZO-1 and claudin-3 were not observed, THY's ability to improve barrier function may involve other pathways. In pig models, THY upregulated gastric mucosa genes related to gut defense (175), suggesting benefits that extend beyond the small intestine. In broiler chickens, THY also alleviated bacterial damage to gut integrity. Moreover, a THY-CAR blend reduced ileum lesion severity during *Clostridium perfringens* challenges while maintaining mucin-2 levels, a protective gut lining component (111). In weaned piglets, THY had limited effects on tight junction proteins under normal conditions (15), suggesting it may be most beneficial under stress or inflammation. On the other hand, in broilers, THY enhances the expression of GLUT2 and SGLT1 nutrient transporters, indicating potential to support nutrient absorption and gut health, which are critical for productivity in animal production systems (19). Overall, THY supplementation shows promise for improving microbiota balance, nutrient absorption, and gut barrier integrity in animals. Its benefits appear most pronounced under inflammatory or stressful conditions, where it may help maintain gut health and productivity, though species-specific effects warrant further investigation.

Additionally, a study suggests that THY may support gut health and development in weaning piglets by modulating gene expression linked to the endocannabinoid system and gut chemosensing pathways (169). THY's influence on CB1 and CB2 cannabinoid receptors, TRPV1 chemosensory receptor, OR1G1 olfactory receptor, and key enzymes in endocannabinoid synthesis and degradation (DGL-α, DGL-β, FAAH) points to a complex regulatory mechanism of gut motility, inflammation, and permeability. The CB1 receptor is primarily involved in regulating gut motility and appetite, whereas CB2 helps modulate immune responses, which may reduce gut inflammation and support overall intestinal health during stress, such as weaning. These effects suggest THY may help pigs to adapt to dietary and microbial changes associated with weaning. On the other hand, THY's modulation of TRPV1 and OR1G1 mRNA levels suggests additional roles in chemosensing and mucosal health. TRPV1, a receptor involved in inflammation and immune responses, may reduce gut irritation caused by dietary shifts or microbiota changes, while OR1G1 aids in nutrient sensing and could optimize nutrient absorption by the gut. By influencing enzymes for endocannabinoid biosynthesis and degradation, THY may balance endocannabinoid tone, potentially enhancing stress resilience and reducing gut inflammation during weaning. Overall, these findings indicate that THY could facilitate a smoother transition to solid food, improving gut health and growth. Furthermore, THY's impact on the balance of endocannabinoids suggests a modulation of the gut-brain axis, potentially reducing anxiety and promoting a more balanced immune response. Clearly, further research is needed to validate these effects across different diets and environmental conditions.

THY supplementation shows strong potential to improve intestinal morphology and gut health across animal models. Studies consistently report increases in villus height, crypt depth, and villus-to-crypt ratios, all markers of nutrient absorption and digestive efficiency, which ultimately support growth and productivity in livestock. In weaned piglets, THY significantly enhances intestinal structure, i.e., resulting in higher villi and improved villus-to-crypt ratios in the jejunum and small intestine (121, 149). These latter changes increase mucosal surface area and improve the absorption of nutrients. Worth mentioning, THY's efficacy can be optimized when combined with other gut health-promoting compounds, such as benzoic acid (BA) (133). Also, in broiler chickens, THY supplementation improves gut morphology, as seen in increased villus height and villus-to-crypt ratios, especially at moderate to high doses (161, 165). As the combination of THY and saponin produced similar enhancements, it is conceivable to hypothesize a synergism between THY and other natural additives (139). Additionally, THY helps maintain intestinal structure under fasting stress, potentially supporting gut integrity in challenging conditions (170). In rabbits and Japanese quail, THY shows comparable effects, promoting villus height and reducing crypt depth, which supports a more efficient digestion (16, 141). Overall, this evidence suggests that THY may enhance nutrient absorption and reduce pathogen colonization in avian species and potentially other target species. In contrast, THY's effects on liver morphology vary. Signs of hepatic stress, such as steatosis and vascular congestion, were observed at high doses in quail, though low necrosis rates suggest a dose-dependent sensitivity (71). Therefore, careful dosing is recommended to balance THY's gut health benefits with potential liver impacts. Finally, THY supplementation in pigeons preserved gut integrity and reduced coccidian stages, supporting its use as a natural alternative to antibiotics in managing infections like coccidiosis (143).

While the effects of THY on intestinal health are well-documented, its potential as a cancer therapeutic, especially for colon cancer, has garnered increasing attention. Although less frequently diagnosed in animals than in humans, colon cancer remains a significant concern in veterinary oncology, particularly among species such as dogs and cats. This malignancy is typically associated with genetic predispositions, chronic inflammation, or exposure to carcinogens. Research on colon cancer in animals is critical not only for improving diagnostic and therapeutic approaches in veterinary medicine but also for providing translational models that enhance our understanding of the disease in humans. A recent study (7) on THY suggests that the compound may significantly reduce tumor markers, such as CA 19-9 and CEA, which are used in clinical settings to monitor colon cancer progression. Additionally, THY's anticancer potential is linked to its ability to induce apoptosis in cancer cells through mitochondrial depolarization, activation of the Bax protein, caspase activation (including caspase-3), and increased ROS levels (36, 73), all of which lead to DNA damage and cell death. This mechanistic action highlights its role as a natural compound capable of modulating cancer cell survival and proliferation. Moreover, THY's effects on oxidative stress and inflammation in colonic tissue further support its role in cancer prevention, as oxidative damage and chronic inflammation are key contributors to carcinogenesis. Histopathological analysis from the same study reveals THY's impact on reducing aberrant crypt foci (ACF) and improving tissue architecture, reinforcing its potential as an agent for preventive cancer therapy.

THY has been shown to exhibit anxiolytic properties in avian and rodent models, effectively reducing stress-related behaviors without impairing motor activity. In quails exposed to restraint stress, THY selectively reduced anxiety without causing motor suppression (176). Similar anxiolytic effects have been observed in rats administered THEO (23). These findings highlight THY's potential as a natural anxiolytic, making it particularly valuable in livestock settings where managing stress is crucial. The anxiolytic effects of THY may be linked to its interaction with the central nervous system, where it likely modulates GABAergic and serotoninergic pathways to reduce anxiety-like behavior. Because THY reduces anxiety without inducing sedation, it could serve as a preferable altersnative to synthetic anxiolytics for stress-sensitive species during transport, weaning, or confinement. Further studies are needed to fully understand its mechanisms and determine optimal dosages across species for broader use in animal care.

Studies have reported THY's dose-dependent anesthetic efficacy in fish, with variations in induction and recovery times. THY's interaction with GABAA receptors suggests unique anesthetic mechanisms that minimize side effects like dependence or tolerance, making it a potentially safer alternative for routine anesthesia (75). In common carp, Yousefi et al. (117) found that THY had slower induction and longer recovery than EUG, suggesting that higher doses or modified protocols may be necessary for quicker effects. Similarly, Boaventura et al. (147) reported that higher doses of THY in tambaqui led to quicker induction but prolonged recovery, emphasizing the need for species-specific dosing protocols. THY's potential as an anesthetic is promising for aquaculture, especially for reducing handling stress during extended procedures. Optimizing its formulation, such as microencapsulation, could enhance THY's bioavailability and accelerate its anesthetic action across diverse fish species. Further studies on THY's neuroreceptor interactions might uncover safer anesthetic pathways for broader veterinary use.

THY also seems to be effective as a wound healing agent in rodents. Incorporated into collagen films or hydrogels, THY enhances wound retraction, re-epithelialization, inflammation control, and collagen deposition, making it a promising natural compound for wound care. In addition, the antioxidant properties of THY are likely to reduce oxidative stress at the wound site, accelerating the healing process. THY-enriched collagen films improved wound retraction and increased collagen density, supporting stronger tissue regeneration (105). Furthermore, a 1% THY-enriched hydrogel accelerated healing in third-degree burns, with faster re-epithelialization and enhanced collagen deposition, suggesting value in treating complex wounds (46). THY's inclusion in collagen-based and hydrogel formulations illustrates its flexibility in wound care applications. These results encourage further testing in larger animal models and clinical settings to confirm its therapeutic efficacy across species. Given its antimicrobial and anti-inflammatory properties, THY holds promise as a multifunctional agent for wound care, especially in complex cases such as burns.

The limited research on THY's effects on bone health includes a study (26) demonstrating its potential as a natural treatment for bone degeneration caused by excessive osteoclast activity, highlighting the need for further investigation. This study shows that THY inhibits osteoclast differentiation and bone loss. *In vitro*, RAW264.7 cells and bone marrow macrophages exposed to THY at concentrations of 10–40 μM exhibited a decrease in tartrate-resistant acid phosphatase (TRAP)-positive cells, a marker of osteoclast differentiation, suggesting interference with osteoclastogenesis. THY also modulates key signaling pathways such as NF-κB, known for its pivotal role in osteoclastogenesis and inflammation. *In vivo*, THY reduced osteoclast numbers and improved bone structure in LPS-induced bone loss in mice treated with 25 and 100 mg/kg. Computer tomography and histological analysis confirmed enhanced bone microarchitecture. These results, though promising, underline the need for more comprehensive research to validate THY's effectiveness in preventing bone resorption. Nonetheless, its potential makes it a promising natural alternative for maintaining bone health in veterinary applications, including aging pets or animals prone to bone disorders.

The only available study on THY's effects on bovine ovarian tissue (129) highlights its potential in supporting follicular activation, maintaining tissue integrity, and enhancing collagen density in *in vitro* cultures. THY's modulation of collagen synthesis and its effect on follicular activation are likely mediated through its interaction with signaling pathways that regulate cell proliferation and differentiation, such as the Wnt/β-catenin pathway. In this study, THY at 400 and 800 μg/mL increased the proportion of morphologically intact follicles, with 400 μg/mL notably promoting primordial follicle activation, essential for early folliculogenesis. Additionally, this dose enhanced stromal cell density and collagen fiber support, suggesting THY's potential involvement in modulating the extracellular matrix (ECM), possibly by affecting the activity of matrix metalloproteinases (MMPs) that regulate tissue remodeling. These changes are crucial for ovarian structure and function. THY's role in follicular activation and structural preservation suggests it could improve the success of assisted reproductive technologies (ART) in cattle, with potential benefits in breeding, conservation, and fertility preservation efforts. Future studies should examine the molecular mechanisms underlying these effects and explore THY's interactions with other reproductive technology compounds.

Finally, evidence from a single study (8) demonstrates that a THY- and menthol-containing gel effectively reduces halitosis in dogs. In a crossover design where each dog received both active and placebo treatments, the gel significantly improved oral odor during the active treatment phase. The antimicrobial and deodorizing properties of THY and menthol are likely to contribute to these results by targeting bacterial populations responsible for malodor, particularly those involved in sulfur compound production. The reduction in halitosis scores, reported by both owners and clinicians, supports the gel's efficacy. These findings suggest that THY-menthol gels could be a valuable addition to canine oral hygiene routines, helping manage persistent bad breath and potentially improving oral health in pets prone to periodontal issues.

In conclusion, this systematic review contributes to the growing body of evidence on THY's multifaceted bioactivity in veterinary medicine, attributed to its broad-spectrum antimicrobial, anti-inflammatory, antioxidant, and antiparasitic properties, among others. THY's efficacy across various animal models demonstrates its capacity to support immune resilience, enhance gut health, and improve overall productivity in livestock. Nevertheless, its safety and effectiveness are contingent upon species-specific dosing and administration forms, as THY's cytotoxicity and pharmacokinetics vary notably across different cell types and animal models. Encapsulation strategies and co-formulations with other bioactive compounds may optimize its therapeutic potential while mitigating risks of toxicity and environmental residues. Given its efficacy in supporting animal health and its potential to reduce antibiotic dependency, THY represents a promising candidate for sustainable disease management in animal husbandry. However, further research into its pharmacodynamic interactions with veterinary drugs and long-term safety in food-producing animals remains essential to maximize its utility as a safe, natural additive.

## Data Availability

The original contributions presented in the study are included in the article/supplementary material, further inquiries can be directed to the corresponding author.
